# Electromagnetism’s Bridge Across the Explanatory Gap: How a Neuroscience/Physics Collaboration Delivers Explanation Into All Theories of Consciousness

**DOI:** 10.3389/fnhum.2022.836046

**Published:** 2022-06-16

**Authors:** Colin G. Hales, Marissa Ericson

**Affiliations:** ^1^Department of Anatomy and Physiology, University of Melbourne, Parkville, VIC, Australia; ^2^Department of Psychology and Clinical Neuroscience, University of Southern California, Los Angeles, CA, United States

**Keywords:** theory of consciousness, electromagnetic field theory, neuroscience, standard model of particle physics, electromagnetic field theory of consciousness

## Abstract

A productive, informative three decades of correlates of phenomenal consciousness (P-Consciousness) have delivered valuable knowledge while simultaneously locating us in a unique and unprecedented explanatory cul-de-sac. Observational correlates are demonstrated to be intrinsically very unlikely to explain or lead to a fundamental principle underlying the strongly emergent 1st-person-perspective (1PP) invisibly stowed away inside them. That lack is now solidly evidenced in practice. To escape our explanatory impasse, this article focuses on fundamental physics (the standard model of particle physics), which brings to light a foundational argument for how the brain is an essentially electromagnetic (EM) field object from the atomic level up. That is, our multitude of correlates of P-Consciousness are actually descriptions of specific EM field behaviors that are posed (hypothesized) as “the right” correlate by a particular theory of consciousness. Because of this, our 30 years of empirical progress can be reinterpreted as, in effect, the delivery of a large body of evidence that the standard model’s EM quadrant can deliver a 1PP. That is, all theories of consciousness are, in the end, merely recipes that select a particular subset of the totality of EM field expression that is brain tissue. With a universal convergence on EM, the science of P-Consciousness becomes a collaborative effort between neuroscience and physics. The collaboration acts in pursuit of a unified explanation applicable to all theories of consciousness while remaining mindful that the process still contains no real explanation as to why or how EM fields deliver a 1PP. The apparent continued lack of explanation is, however, different: this time, the way forward is opened through its direct connection to fundamental physics. This is the first result (Part I). Part II posits, in general terms, a structural (epistemic) add-on/upgrade to the standard model that has the potential to deliver the missing route to an explanation of how subjectivity is delivered through EM fields. The revised standard model, under the neuroscience/physics collaboration, intimately integrates with the existing “correlates of-” paradigm, which acts as its source of empirical evidence. No existing theory of consciousness is lost or invalidated.

## Introduction

2020 marked the 30th birthday of the modern form of the empirical science of consciousness introduced by Crick and Koch’s influential 1990 “*Toward a neurobiological theory of consciousness*” (Crick and Koch, 1990). The science of consciousness, or the science of phenomenal consciousness, as David Chalmers described it (Chalmers, 1996), is the scientific account of the 1st-person perspective (1PP), a perspective that delivers subjective experience (subjectivity) or “what it is like to be us” (Nagel, 1974). Throughout this article, we also interchangeably employ the term P-Consciousness (Block, 1995) to refer to the 1PP.

That moment in 1990 has a threefold significance. First, it marked a transition of the science of P-Consciousness into the physical sciences. Prior to this time, it could be career trouble for neuroscientists to directly attend to P-Consciousness (Koch, 2019). Mainstream neuroscientists confined themselves to the neurobiological account of nervous system function, its disorders, and their treatment. Post-1990, however, the swelling ranks of neuroscientists funded in the science of P-Consciousness have made remarkable progress.

Second, neuroscientists embraced a categorically distinct, novel explanandum unprecedented in any physical science: the 1PP of the nature under consideration. No other physical science tackles this. It tends to be under-acknowledged that on behalf of all physical sciences, and alone, neuroscientists have entered the realm of the novel explanandum that is the 1PP. The reason for this solitude is that neuroscience’s central target, the human brain, is the only natural context with an empirically proven, accessible instance of a 1PP. Its existence has led to the science of consciousness: we know that somehow, some of the details found in “being” the activity of this vast neural/glial complex is highly correlated with the details of P-Consciousness. Neuroscience alone has this evidence base within its explanatory ambit.

Consider that no geologist is currently required to or is able to account for what it is like to be a rock, from the perspective of being a rock made of atoms from the same table of elements used in a brain. Neuroscience that successfully explains the origins of P-Consciousness in brains will consequently and incidentally facilitate, ultimately, an equally proved scientific basis for what it is like to be a rock. This is not any kind of claim that rocks are conscious. This is about being able to say something scientific about the consciousness of a rock. A claim “*It is not like anything to be a natural rock*,” originating in a future mature science of consciousness, will be a formal scientific position, not merely an intuition. In this future, all scientists will require enough training in the science of P-Consciousness to accurately conceive of how it impacts their purview.

The third feature of the 1990 moment was Crick and Koch’s prototyping of a way to normalize the science so that the peculiarities of the novel explanandum could yield to the familiar and trusted empirical methods of neuroscience. This normalization explicitly used the term “correlates” in an empirical method (Crick and Koch, 1990). Their proposition became the “Neural Correlates of Consciousness” (NCC) empirical paradigm, which triggered an extensive literature examining NCC and the idea of “correlates” as an empirical evidence approach that is still ongoing and productive (Salthe, 1985; Crick, 1994; Chalmers, 2000; Metzinger, 2000; Rees et al., 2002; Farber, 2005; Mormann and Koch, 2007; Rees and Frith, 2007; Velmans and Schneider(eds), 2007; Lau, 2008; Molyneux, 2010; Aru et al., 2012; de Graaf et al., 2012; Neisser, 2012; Dehaene, 2014; Axelrod et al., 2015; Tsuchiya et al., 2015; Koch et al., 2016; Mashour and Hudetz, 2018).

A technically accurate generic depiction of the science of consciousness is “*an ongoing attempt to locate and describe the ABC-correlates of consciousness.*” Crick and Koch set the first ABC to “neuronal.” All scientific theories of consciousness can be cast in the “ABC-correlates” format. A non-exhaustive list is ABC = “*behavioral*” e.g. (Tononi and Koch, 2015), “*global workspace*,” “*integrated information*,” “*computation*,” “*thalamocortical loop*” e.g. (LaBerge and Kasevich, 2007; Winters, 2021), “*oscillatory/resonance*,” “*mathematical,”* “*quantum mechanical*” *-correlates of consciousness*. Note that this idea can be extended to ABC = “*philosophical*” correlates such as “*functionalism*,” “*physicalism*,” and many others. In each case, to suggest an “ABC Theory of Consciousness” is to describe the operation of a brain from an ABC-correlates perspective. The idea is that researchers with enough reproducible empirical evidence of “ABC-correlates of P-Consciousness” can claim to have accounted for P-Consciousness in a familiar, well-traveled manner.

However, decades of experimental work have revealed that even if a particular ABC becomes strongly evidenced, the 1PP will still not be explained. We find ourselves frustrated and forced to admit that ABC-correlates all suffer the same fate as explanation: they do not explain why the particulars of an ABC necessitate a 1PP. We are simultaneously all aware of why this happens: “*However, correlations by themselves cannot supply explanations, they can only constrain them*” (Seth, 2009). In every ABC case, the 1PP somehow just “happens” *via* a mechanism or principle that is not delivered by knowledge of the ABC-correlates. Instead of an explanation, we find ourselves in possession of a collection of collaborating ABC “parts,” whose individual connection to a mechanism of a 1PP is lacking, that somehow manages to create a “whole” that delivers it. Later, this explanatory failure will be formally classified as “strong” or “radical” emergence, in contrast with the normal kind of “weak emergence” that is regarded as a successful explanation in science elsewhere.

This quandary is another way of portraying the “hard problem” of consciousness as described by Chalmers (1996), in which he recognizes that P-Consciousness is not explained merely by describing matter (ABC). Why should matter behaving “ABC-ly” be mysteriously tagged with a first-person perspective? Why do we expect the simple enumeration of ABC-correlates to deliver an explanation?

We can more deftly touch the origins of our frustration by an analogy using Newton’s 2nd Law, *F* = *mA*, when notionally used to capture “what it is like to be mass *m*.” Stimulated by force *F*, if the responding acceleration term *mA* could “talk” to deliver the “*mA-correlate”* of an extra explanandum, the P-Consciousness of mass *m*, then the science is structured identically to the ABC-correlates paradigm. Nothing in the statement *F* = *mA* explains *why F* = *mA*. In exactly the same way, nothing in the *mA* term explains why mass *m* has P-Consciousness. The formula *F* = *mA* could be described as the “force correlate of acceleration.” It was constructed because *F* and *m* behavior are directly evidenced (even if *via* solid inference) in the normal manner of scientific observation (originating as the contents of the consciousness of the scientific observer). In the “*mA-correlate”* of the P-Consciousness of mass *m*, we cannot evidence both sides of the relationship in the familiar way. The process actually delivers the correlate of a P-Consciousness *report.* In what amounts to a highly curated form of hearsay evidence, reports can be explicit (“report”) and implicit (“no report”) (Tsuchiya et al., 2015). Because of the indirectness and non-uniqueness of the evidence, we cannot conclusively argue to have encountered the “correct” ABC-correlate. To make that crucial argument a cogent argument is impossible because it presupposes accurate knowledge of the 1PP that the ABC is somehow expected to deliver. That makes the expectation of uncovering a real explanation (the underlying 1PP mechanism/principle) from ABC-correlates optimistic at best. This is the now 30-year-old, familiar struggle that all ABC-correlates scientists face daily at the coalface of empirical work and critical argument in respect of the origins of P-Consciousness (Pitts et al., 2014; Storm et al., 2017).

The search for the elusive “smoking gun” ABC-correlate has certainly been worth the effort. It has revealed most of what we now know about P-Consciousness. It has been a very successful program of work. However, the “explanatory cul-de-sac” is a deeply unsatisfactory state for our knowledge of consciousness. It continues to locate practitioners out of reach of an empirically proved full explanation, leaving us all in the grip of strong emergence. This is the paradoxical presentation of the current operational structure of the science. After 30 years, these observations deliver us the license and an obligation to explore the possibility of a way to transcend the strong-emergence cul-de-sac. This article is a result of that exploration.

In what follows, we do not deliver the “correct” ABC theory of consciousness or a set of modifications to an existing ABC. One of the existing ABC, or perhaps a combination thereof, is likely to be “right.” This article is agnostic in that regard. Our interest is in how it is to be conclusively *proved*. It is the inconclusive evidence basis, in the face of the unique explanandum that is the 1PP, that we address here. That is, on behalf of all ABC, this article targets the reason for the explanatory cul-de-sac and what to do about it. In what we have called an “electromagnetic turn” in the science of consciousness, we demonstrate that it is in the science of consciousness acquiring its mature operational structure that leads all ABC out of the explanatory cul-de-sac. In the process, all (top–down) ABC get their long-sought connection to explanation and proof in (bottom–up) fundamental physics. The result is delivered in two parts.

In Part I, we first explore the brain from a fundamental physics perspective (the standard model of particle physics). It reveals the brain to be an intrinsically unitary electromagnetic (EM) field object, seamlessly impressed throughout and beyond the space occupied by the brain’s cellular componentry, from the atomic level up. We include a review of the anatomical membrane-scale origins of endogenous EM field expression by brain tissue (see Supplementary Material A). This is followed by an analysis of six classes of ABC-correlates theories of consciousness, confirming how each class locates itself in the strong emergence cul-de-sac. The brain’s specialized complexity in EM field expression distinguishes it from other organs (such as the liver and the heart) that are also EM field entities from the atomic level up. The consequence is that there is only one natural, fundamental physics correlate of P-Consciousness: EM fields as “electromagnetic correlates of consciousness” (EMCC). ABC-correlates neuroscience has, in effect, implicitly proved that (bottom–up) EM is the ultimate origin of the 1PP for all (top–down) ABC-correlates. This has the consequence of moving EM field expression by brains to center-stage in the science of consciousness, thereby positioning neuroscience in the heart of fundamental physics. This is the first result.

In Part II, inspired by the Part I analysis of six classes of ABC-correlates, we deliver the second, speculative result. It reframes the science of P-Consciousness into a neuroscience/physics collaboration charged with accounting for how it is that standard model EM fields have, within them, the potential for a 1PP, and what EM is doing when it delivers it in a brain. Part II is a preliminary/introductory discussion outlining how, in the EM basis of all ABC, neuroscience and physics communities may collaborate to discover how EM fields acquire the potential for subjectivity that neuroscience has proved must exist within them. In that final EM account, all ABC-correlate theories of consciousness gain, from fundamental physics, a common link to an explanation. Physics benefits in acquiring, from neuroscience, a route to an explanation of the scientific observer. Together, the two science communities have the potential to build a viable bridge over the explanatory gap (Levine, 1983; Van Gulick, 2018) that offers hope for a solution to the “hard problem”. The practicalities of the implementation and empirical proof of the proposal are described in general terms as the beginning of an ongoing discourse that can guide us into the future.

## Part I: ABC-Correlates of Consciousness Are Electromagnetic Correlates

We now detail how the following claim is a natural consequence of the standard model of particle physics:


Claim C1=All “ABC correlates of consciousness” are actually                       “electromagnetic correlates of consciousness”                       (EMCC) in ABC guise.

Claim C1 is not a theory of consciousness. C1 merely recognizes that whatever the ABC, it is ultimately implemented by some subset of the EM field behavior comprising a brain. To proceed with precision, let us specialize the context of claim C1 to humans. As already advised in the introduction, human 1PP is the only proven, accessible instance of it known to science. The human 1PP has led us to the need for and development of a science of consciousness. The human 1PP thus becomes the primary explanandum of the science. Extending the explanation to include the 1PP of non-human fauna, flora, and artificial/machine consciousness can be left to a separate discussion because it changes nothing in relation to the validity of C1, which is based on simply taking a fundamental physics perspective of the human brain. Note that the fundamental EM basis of the 1PP has previously been examined from specific perspectives (Barrett, 2014; McFadden, 2020). *Via* C1, this article extends the work to a full evaluation including its generalized implications.

### The Standard Model of Particle Physics

**FIGURE 1:**
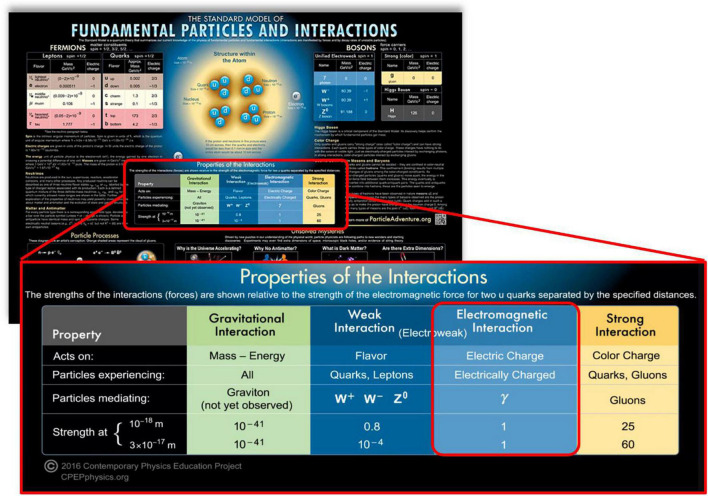
A popular representation of the standard model of particle physics in which we find the four fundamental forces, within which is the electromagnetic force. For further information, a useful starting point can be found in Aitchison and Hey (1984), Halzen et al. (1985), Cottingham and Greenwood (2007), and Griffiths (2020). Credit: the model can be purchased in many forms from the non-profit “Contemporary Education Physics Project” (https://www.CPEPphysics.org).

The Standard Model of Particle Physics (SMPP) best (but not yet perfectly) describes the physical basis of everything found within the space comprising our universe, along with the properties of space itself (Cottingham and Greenwood, 2007; Rich, 2010). The SMPP has four quadrants (see Figure 1) covering four fundamental forces: EM, strong-nuclear, weak nuclear, and gravitation/inertia. This presents us with a fundamental ontology of forces based on the fields that manifest them. This list is exhaustive. There are no others known to exist. Claim C1 confines us specifically to the highlighted Figure 1 EM field quadrant of the standard model [for physicists it is known as the U(1) symmetry group].

The SMPP delivers a stark shift in neuroscience’s comprehension of the true nature of the atomic basis of our biosphere. The reason for the C1 confinement to EM fields as originating the 1PP is a very simple one: In the context of the atomic basis of everything relevant to life in our biosphere, there is literally nothing else to hold accountable for the 1PP because there is nothing else there to choose from: *It is all EM field*. Choosing from a list of one item is a very simple and attractive choice. It is the job of the rest of Part I to demonstrate how the SMPP proves C1, and how this has been the case ever since the standard model was assembled half a century ago. What has changed, paradoxically, is that the SMPP news will finally reach neuroscience in the context of a 30-year-old empirical, physical science of P-Consciousness.

The SMPP picture of “matter” made of atoms involves atomic nuclei and electrons as the collaborating particles that comprise the material (atomic) basis of our biosphere. These are vanishingly small, punctate containers of all the deep driving originators and constraints leading to the atomic basis of the biosphere. Synergy between atomic nuclei and electrons is fundamentally defined by their electromagnetic properties, the most dominant of which is their electric charge and magnetic moment (spin) source content. Everything else about the particles (such as their associated mass), in our context of interest, is secondary.

Consider a rough and conservative estimate of the spatial occupancy by the interior of electrons and nuclei, as a proportion of the space attributed to being occupied by a complete, typical atom from the table of the elements. If we divide the spatial occupancy of a single atom into 15,000 parts, the amount of space occupied by electrons and nuclei is a fraction of 1 part (Kitchener and Hales, 2022). Contained within that part are all the charge and spin sources expressing EM fields that intimately interrelate in the manner that stabilizes an atom that, in ionized form, can then impress EM fields on space at distances far greater than the size of the atom. In this way, the position and motion of charge and spin, existing in space at the vanishingly small level of the interior of atomic particles, literally create the EM field that exists in the space outside atoms, manifesting the forces that have us regarding it as “material” or a “substance” behaving “physically.” EM fields are the ultimate origin of the forces that create atoms and hold them together to make molecules and higher-level structures (this organizational hierarchy is detailed below).

More generally, at and above the level of the atomic particles, the Figure 1 SMPP tells us that the familiar atomic basis of the material of our biosphere is entirely comprised of only three things: First is space itself. The second originates in the SMPP EM field quadrant, which tells us we have large EM fields originating from the charge/spin source content within the interior of atomic particles. The third originates in the SMPP gravitation quadrant, which tells us we have a gravitational field originating in the mass intimately entangled with the EM (charge/spin) sources *inside the same atomic particles*. Figure 1 also tells us that the gravitational field can be regarded as functionally inert at the level of the biosphere contents (things like humans and our brains). This is because the gravitational force is at least 16 orders of magnitude weaker than the EM field force. While the mass delivers inertia into the Newtonian transport dynamics motivated by the EM field forces that charges/spins mutually experience and impose on each other (the Lorentz force – see Supplementary Material A), this is distinct from the gravitational field forces produced by the mass component of the atomic particles. For all intents and purposes here, the space occupied by a brain is therefore effectively entirely permeated by nothing but EM fields.

This cursory appreciation of basic SMPP facts is, in fact, loaded with a fundamental challenge. As scientists, we must face the rather confronting fact that our own standard model is telling us that, for all practical purposes in the science of P-Consciousness, we *are* electromagnetic field objects in our entirety. As is a car, a computer, lunch, a pile of dirt, a tree, your dog, steam, and the air we breathe. When we use the words “physical” or “material” in the natural context of the brain’s delivery of a 1PP, these words refer to the supra-atomic scale EM fields impressed on space by atoms. In a quest to understand the 1PP that arises from “being” made of atoms, to the extreme levels enumerated above, there is nothing else left to hold accountable for the origins of the 1PP but EM fields because there is, effectively, nothing else there to be found but EM fields.

Fully engaging and substantiating this change in perspective involves details that it is the job of the rest of Part I to assemble. We do not intend to deliver anything but the mundane, long-proved empirical reality delivered by the SMPP. There are no new facts here. We simply engage more fully in what physics tells us of the brain and how it relates to a potential scientific account of the origins of P-Consciousness.

For completeness in an understanding of how EM may ultimately be understood to originate the 1PP *via* C1, we now consider the remaining three quadrants of the standard model: the strong-nuclear, weak nuclear, and gravitation/inertia quadrants. The strong and weak nuclear forces are exquisitely localized within the nucleus of atoms, holding the nucleus together. Together, these three quadrants create and maintain the structure and dynamics of the atomic basis of all the members of the table of the elements, thereby creating and stabilizing the EM field quadrant as expressed by atoms. In effect, they form an intra-atomic constraining envelope for EM fields to work at the scale of life and consciousness.

That said, C1 does not entail any presupposition that the intimate entanglement of all four quadrants, at the subatomic and deeper levels, has no role in some aspect of the production of P-Consciousness. Put simply, the core fundamental claim of C1 is that the EM field quadrant has *primacy* in the establishment of P-Consciousness for human brains, including its array of qualitative kinds and their degrees. C1 is upheld even when all four quadrants of the standard model may ultimately be proved to be severally necessary and only then jointly sufficient to produce P-Consciousness. Note that the three subsidiary quadrants are physically contained within, and expressed by, the atomic layer of the natural matter hierarchy to be discussed in the next section. Therefore, any account of the EM quadrant involving atoms (and therefore their components) implicitly entails the presence of the other three quadrants. To that extent, in reality, C1 invokes a necessity for all four quadrants, while claiming that it is ultimately the electromagnetic quadrant that physically results in P-Consciousness being associated with brains. A practical note: EM field theories of consciousness that partition electromagnetism across a supra-/infra- atomic/molecular level boundary exist and are consistent with C1 e.g., (Poznanski et al., 2019; Keppler, 2021).

### C1 as a Consequence of a Natural Containment Hierarchy

**FIGURE 2:**
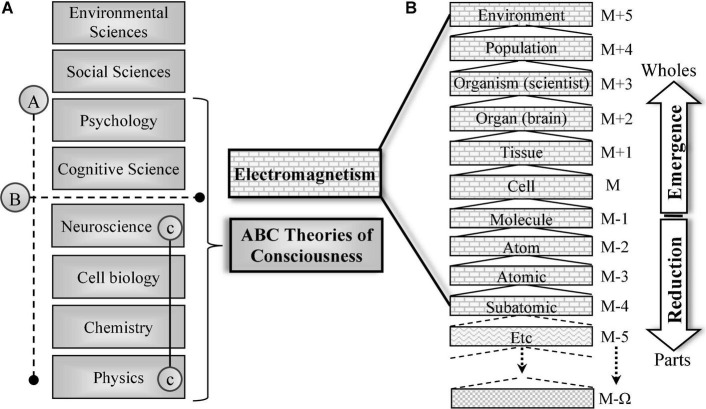
**(A)** The organizational hierarchy of sciences in the lineage relevant to the study of the brain, consciousness, cognition, intelligence, and behavior. Line A shows the hierarchy of the sciences tackling consciousness. Line B depicts the upper boundary of the physical sciences. Line C shows the connection between neuroscience and the standard model of particle physics that is central to Parts I and II. **(B)** The natural hierarchy identified by scientists beneath line B. Each layer of description is constructed from within the hierarchy by scientists within layer [M + 3]. The specific case shown leads deep into the ultra-structure of the notional brain of the particular observing/describing scientist. Each layer is a collection of weakly emergent “wholes” comprised of members of all the layers below. Based on Hales (2014, Figure 10.1). See also Feinberg (2011, Figure 7) and Bongard and Levin (2021, Figure 1) and hierarchy theory in general (Koestler, 1967, 1978; Grobstein, 1973; Pattee, 1973; Simon, 1973; Allen and Starr, 1982; Salthe, 1985; Ahl and Allen, 1996).

Switching to a transdisciplinary view, as a fundamental shift in perspective, is key to understanding the EM origins of P-Consciousness. Consider Figure 2A line A, which depicts all existing ABC theories listed in roughly science-disciplinary order. The physical sciences shown in Figure 2A below line B have discovered the nested containment hierarchy of our biosphere shown in Figure 2B. It depicts the hierarchy seen on a generic descriptive trajectory leading deep into the excitable cell tissue *of a scientist*. Take a moment to compare it with a very specific descriptive trajectory into the brain (say, into a particular mitochondrial DNA codon), or a descriptive trajectory taken through a rock, a kidney, a computer, a tree, or a star.

The Figure 2B descriptive trajectory has been deliberately chosen to highlight the position of all scientists within the hierarchy at the organism layer [M+3]. This can be viewed as a specialized sample from the population of humans included in Figure 2B layer [M+4] that are contained within our biosphere, the environment layer [M+5]. The symbol M serves merely as a reference point to ensure consistency and accuracy.

The overall height of the hierarchy is artificially limited to suit our context. It does not include higher levels of containment, such as a planetary system. The accretion of Figure 2B hierarchy layers arises in synergies between aggregated members at a particular layer (spatiotemporal scale). Within a given layer, “horizontal” aggregates of members (parts) of deeper layers form qualitatively new composites (wholes). For example, atoms form molecules and they jointly aggregate to form cellular organelles. These jointly form whole cells, and so forth. It is explaining these qualitatively novel, persistent organizational structures that attracts the attention of Figure 2A scientists.

The Figure 2 nested containment hierarchy perspective appropriately grounds our activities as scientists in our pursuit of a scientific understanding of the natural biosphere hierarchy, from within that biosphere hierarchy, by use of properties acquired by being literally made of what the biosphere hierarchy is made of.

Describing the observed apparent structure at some point in the hierarchy has traditionally located us in our chosen science discipline. The labeling of a layer’s appearance by scientifically behaving humans is a mere human abstraction of the layer’s characteristics (such as thermodynamic, informational, cognitive, and so forth), including influences from or properties of the lower layers. If you deleted (in the sense of “de-organized”) any layer below M, for example, the entire hierarchy disappears from that layer upwards. For example, deleting all atomic particles deletes atoms, molecules, cells, and so forth, all the way to the containing environment. In these cases, none of the deletions eliminate the lower levels, including sub-atomic particles, space, and so forth. This fact reveals the existence of a powerful *vertically* acting system of constraints that is not within the ambit of any individual scientific discipline. This system of constraints operates through the entire hierarchy, from top to bottom e.g. (Feinberg, 2011, Figure 7).

Next, from a position within the environment layer [M+5], we take in the inward, transdisciplinary perspective “down” the hierarchy. The Figure 2B hierarchical whole/part decomposition takes a descriptive vector indefinitely deep into our nominated scientist’s brain tissue layer [M+2] at finer and finer spatiotemporal scales. Note that to “be” a scientist’s brain at layer [M+2], by definition, includes everything in all deeper layers. If there are a thousand layers of nested structural containment hierarchy beneath [M+2] then we human scientists are “being” all of them. Note that the hierarchy is depicted as ending at some unspecified deep layer signified as [M-Ω], the possibility and nature of which is beyond the scope of this article. It does not impact C1.

In representing the natural hierarchy in this way, the phrase “fundamental physics” acquires the practical meaning needed to support C1 and interpret its implications. The SMPP is a set of empirically proven formal descriptions, beginning around layer [M-5], that covers a vast range of exotic entities, most of which are not relevant to the science of our primary explanandum, human P-Consciousness, because they are uninvolved in human anatomy or physiology from the atomic level up.

The EM field basis of our chosen vector through the hierarchy is indicated by the “electromagnetism” layers with the brick background in Figure 2B. It arises at the organizational level [M–4] (protons, neutrons and electrons). Accreting (vector-superposing) layers of electromagnetic fields, starting at the deepest levels of quantum electrodynamics, form the fundamental basis of everything above the subatomic layer [M–4], culminating in the creation of our biosphere [M+5], the containing environment of our notional scientist in a natural setting. The hierarchy (as previously described) is, in effect, entirely EM field from the atomic level [M–3] (electrons and nuclei) up.

Consider now the specific context of interest here. Those of us studying the science of P-Consciousness are located with all other scientists as particular “organisms” within layer [M+3]. The scientific outcome is, in effect, a scientific account of the scientific observer. Applied to the context of the acquisition of objective scientific evidence (originating as “contents of a scientist’s P-Consciousness”), an explanation of the observer has the consequence of explaining how scientists can “scientifically observe” the biosphere from our layer [M+3] position within the hierarchy. The Figure 2 transdisciplinary view thereby reveals the science of consciousness as part of a science of how we can do any science at all. In the ABC-correlates context, this literally makes us scientific observers trying to explain P-Consciousness (an ability to scientifically observe) through the use of scientific observation (P-Consciousness). Figure 2 thereby demonstrates the logical flaw of “question-begging” at the heart of the recognized difficulties with the process of explanation within the ABC-correlates of consciousness paradigm: We are using observation (of ABC-correlates of a consciousness report delivered *via* the 1PP of a presupposed human scientist observer) to account for how we scientists can observe anything at all. The result is that, because of the way a presupposed 1PP is used to source all scientific evidence, science is essentially rendered voiceless in respect of an explanation of the 1PP.

When it comes to the science of P-Consciousness, we must all face the vertical hierarchy of Figure 2B and our position within its layers. It is the complete hierarchy that delivers P-Consciousness, not any abstractions of it (such as the ABC of the introduction) created by a practitioner making a horizontal slice, thereby abstracting-away the fundamental EM basis of P-Consciousness that arises in the entirety of the hierarchy. To prevail over this unique and unprecedented explanandum in science, we must all shift our perspective from a horizontal discipline-centric reductive view to a vertical transdisciplinary holistic view. In doing so, we all encounter fundamental physics — in this case, the fundamental physics of EM in the standard model.

Hierarchically organized EM fields are still just EM fields. However, their intimately nested hierarchical structure raises the possibility that, depending on how layers are organized, fundamental properties of consciousness such as “unity” (Cleeremans and Frith, 2003; Bayne, 2010) or “symbol-grounding” or “binding” or “combination” (Harnad, 1990; Treisman, 1996; Revonsuo and Newman, 1999; Roskies, 1999; Singer, 2001; Chalmers, 2016), inherited at layer [M–4], can potentially be conserved, inherited and incorporated in higher organizational layers. For more detail on these nuanced aspects of consciousness and EM’s natural suitability in accounting for them see Kitchener and Hales (2022). In relation to EM’s natural solution to the combination problem, for example, the “inheritance” is literally manifest in the layered accretion (vector field superposition) of EM fields, where qualitatively novel emergent 1PP wholes can be traced back to vectorially superadded EM field parts. Insofar as any brain property may be inherited in the deep layers and then assembled with more complexity as the layers accrete as a coherent unity, the Figure 2B vertical direction is the ultimate origin of anything that can be claimed to be “emergent” in the hierarchy. That accreted/collective inheritance may then act with an emergent influence and have functional implications. The concept of emergence is formalized below.

It is in Figure 2B cranial central nervous system organ layer [M+2] that we can conceive of brains as an exotic solid entirely comprised of EM field phenomena expressed as a deeply nested containment hierarchy. It is, therefore, only a transdisciplinary perspective that can fully account for P-Consciousness as a collective property of layer [M+1] tissue, regarded as an EM field object. Layer [M+1] is where the EM field system impressed on space by brain tissue acquires its fully detailed form, including all properties inherited by the constraints, drives, and properties of the deeper layers. The EM field system is impressed on space with a spatial organizational resolution of 7–8 orders of magnitude, from the nm scale of the cell membrane to cm at the tissue scale.

When sampled within scientific disciplines, we find that scientists tend not to answer the question “*What are humans/human brains made of*?” either consistently or with technical specificity. In day-to-day science, this inconsistency simply does not matter much. However, we have now seen how fundamental physics has answered the question: Humans are made of the EM fields expressed by atoms based on the subatomic-level electric charge and magnetic spin sources that originate them. In principle, that answer should suffice, regardless of one’s disciplinary standpoint. Differently organized EM fields are still EM fields, just as two very different kinds of wall can be made of the same bricks, and when each wall is finished, the bricks are still just bricks. The EM field of different sources intimately sum, through vector superposition, into a single, unified EM field whole in a way that bricks do not, but the result has the same interpretation. The origins of P-Consciousness must ultimately rest on our fundamental composition/whatever it is we are made of. At the heart of the answer is the fundamental EM field basis of cranial nervous tissue. Somehow, “being” these exquisitely detailed EM fields, manifest by an atomic substrate, delivers P-Consciousness, however mysterious the connection may be.

### C1 in Measurement and Control

We now examine the scientific evidence collected in empirical work published in the science of consciousness at the various levels in the Figure 2B hierarchy. In the context of a brain, within the hierarchy shown, we can enumerate the types of measured data and their method of acquisition. First, consider raw counts. If we are studying diffusion processes, we are, in effect, looking at spatiotemporal counts (thermal concentration dynamics) of what are ultimately electromagnetic objects randomly flying through space and colliding with each other.

Consider the ubiquitous measurements known as Local Field Potentials (LFP) (see Einevoll et al., 2013), the Electrocorticogram (ECoG), the Electroencephalogram (EEG), and the Magnetoencephalogram (MEG) (Buzsaki et al., 2012; Obien et al., 2015). These are all measurements of spatially averaged, time-sampled/temporally averaged electric and magnetic field properties at a nominated spatiotemporal scale. Each of these kinds of measurement bears witness to the EM nature of the measured object. MRI/fMRI, scanning and transmission electron microscopes, atomic force microscopy and all forms of probes, actuators and stimulators are also an EM interaction with the studied material.

Insofar as brains are able to perform sensory measurement, the same concept applies. All of the sensory modes are, in the end, EM field phenomena, even those that are thought of as purely chemical or mechanical. When we touch something with our finger or another appendage (or, more generally, when atoms of any kind collide), at the atomic level, EM fields interact with EM fields. That is what “touching” is. The process of sound waves impacting sensory hairs in the cochlea is also ultimately an EM field interaction. Sound transmission occurs through the propagation of phonons (disturbances in the EM field system at the atom level of Figure 2 layer [M–2]). We tend to think of sound as a “mechanical” property. In reality, the “mechanical” descriptor is merely a label we apply to what is actually an EM phenomenon. Phonons are bosonic (Ashcroft and Mermin, 1976, pp. 780–783; Feynman, 1976, p. 159), originating naturally within the EM quadrant of the standard model of particle physics through their atomic-level propagation mechanism.

When it comes to the artificial control of the operation of brain signaling, all the various forms of it involve the exogenous application of EM fields. Consider transcranial magnetic and electric stimulation (TMS/TES) or intracranial electrical stimulation (Raccah et al., 2021). These are clearly and entirely the topical application of EM fields to influence the brain’s endogenous EM field system either for exploratory or clinical purposes. In the same context, brain tissue surface and penetrating electrodes also function by delivering EM field system influences and, similarly, acquire their effectiveness *because* of the EM field basis of the brain’s endogenous signaling systems. Another more recent arrival in this area is transcranial ultrasonic stimulation (TUS). This, too, is an EM phenomenon for the reasons stated in the previous paragraph. Introducing chemicals into the brain is also the introduction of EM field phenomena. Surgery is also an EM field disruption using the EM field of surgical instruments.

In this way, all sensory/motor action within a brain, and all scientific measurement and control applied in the science of P-Consciousness, implicitly involve EM field properties in the chosen context. This empirical reality undergirds C1 and the EM field basis of P-Consciousness, with these diverse phenomena ultimately becoming the measured electromagnetic correlates of consciousness (EMCC) cited in C1. Essentially every measurement ever made in support of any ABC theory of consciousness is also an EMCC acting in support of an EM field theory. C1 thereby serves to reinforce the fact of the EM basis of all brain phenomena as something the science has been implicitly involved in all along.

### The Hierarchy and Weak/Strong Emergence

For the purposes of completing our examination of C1 and connecting it with the processes of explanation in the science of P-Consciousness, here we calibrate our understanding of emergence and how it operates in the natural hierarchy of Figure 2B. The brain is a thermodynamically open, far-from-equilibrium, non-linear, non-stationary, self-assembling, self-organizing complex dynamical system with power-law dynamics (e.g., Kitzbichler et al., 2009; Fingelkurts et al., 2013; Zare and Grigolini, 2013; Cocchi et al., 2017; Tagliazucchi, 2017) based on the Figure 2B nested physical containment hierarchy of fundamental EM field activity impressed on space by excitable cells forming brain tissue organized in the manner detailed in Supplementary Material A.

Two technical categories apply to complexity expressed by hierarchical systems. The first category, “weak-emergence,” signifies a collective behavior that is not obviously related to any individual part/component, yet is a “whole” that is, in-principle, predictable and explained by a sufficiently detailed exploration of groupings of well-understood, explained, and predictable parts. The containment hierarchy in Figure 2B is a nested hierarchy of natural “weakly-emergent” objects described by scientists in Figure 2A.

The second category occurs when a property of complexity in nature defies such prediction and cannot be found in a description of collective behavior. It is a failure of explanation called “strong/radical emergence” (sometimes “magical emergence”; e.g., see Rosen, 2012, p172). It occurs when a property is so completely unexpected and unpredicted that its presence seems magical and signifies that something is missing in our knowledge of the natural world (Baas, 1994; Bedau, 1997; Van Gulick, 2001; Corning, 2002; De Wolf and Holvoet, 2005; Chalmers, 2006; Clayton and Davies, 2006; Kim, 2006; Stepney et al., 2006; Hendry et al., 2019; O’Connor, 2020).

We now note that the transition from strong to weak emergence is a fundamental feature of the process that science experienced when deconstructing the natural biosphere into the layered descriptions shown in Figure 2B. In Figure 2B this process has been labeled as “reduction.” Before the science was completed, every progression in scientific understanding started as a mystery: a question unanswered. Molecules were mysteriously related to atoms. Atoms were mysteriously emergent from what turned out to be their subatomic constituents. Higher up, we find the mystery of the strongly emergent flight of bumblebees, which turned out to be a weakly-emergent property of turbulence. The story of science is a multiplicity of singular moments of transition, in the vertical direction, from initially strongly emergent wholes that were eventually reduced to weakly emergent properties of sufficiently well-understood parts. In each case, it was scientific practitioners operating at a particular descriptive level in Figure 2A that curated the transition from strong to weak emergence in the day-to-day operation of science. It is the job of this section to clearly articulate, *via* C1, how the location of ABC-correlates of a 1PP does not transform the 1PP from strong to weak emergence.

### The Ultra-Scale Origins of Excitable Cell Tissue-Level Electromagnetic Fields

To complete the picture of the EM field nature of brain tissue under C1, a comprehensive overview of the expression of the EM field basis of the familiar, dominant endogenous EM field system involved in brain tissue intercellular signaling has been included in Supplementary Material A. It describes how Figure 2B layer [M] excitable cell behavior (in our Figure 2B notional scientist’s brain) expresses (literally is) a pair of unitary fields: an electric field **E** and a magnetic field **B**, each expressed with seven to eight orders of magnitude of structural resolution (from layer [M–2] atomic-dimensions up). These two fields, in static and dynamic forms, pervade all the space occupied by the brain, spilling out into the space around the organ layer [M+2]. The familiar endogenous EM field system of the brain originates in the nanometer-scale (sub-cellular) action of the membrane of neurons and glia. To comprehend C1, simply recognize that to place an atom in space is to place an EM field system in space. When an atom or molecule has an imbalance of charge it becomes an ionic net source of electric field system in space. In the formal sense of Maxwell’s equations of electromagnetism, charges positioned in space become a source of electric charge density expressing electric field. Moving charges become a source of current density that introduces a dynamic magnetic field. Together these two systems of sources produce the dominant static and dynamic **E** field and a purely dynamic **B** field that inherit the Figure 2B tissue ultra-structure in their layout in space.

Further elaboration of the details of the EM field source system is included in Supplementary Material A. Everything needed to articulate the case for EM field as the ultimate origin of P-Consciousness exists in well-known cell biophysics interpreted from a fundamental physics perspective. Under C1, when ABC-correlate researchers at any Figure 2A level mentally step into the Figure 2B hierarchy, turn, and look down into the deep vertical structure, this is what ABC-correlates ultimately look like: a finely expressed collection of EM field activity impressed on space with an atomic-level resolution by excitable cells in the manner of Supplementary Figure A.1. It is posited here that “being” this EM field behavior delivers the 1PP, albeit for reasons not understood. As outlined in the section on the SMPP, this is a result of the tissue literally “being” an EM field system and the fact that there is nothing else to hold accountable for a 1PP. Until this possibility is empirically refuted, it is a reasonable basis for directing research into the 1PP.

The C1 convergence on EM fields and the Supplementary Material A depiction of the origins of EM in brain tissue reveal an anomaly in neuroscience practice. Neuroscience completely lacks the inorganic (*in silico*) replication of the (Supplementary Material A) kind of EM fields expressed by cell membrane. The creation of chip materials able to express EM fields structurally identical to those produced by neurons can be used to construct artificial neurons that replicate neuron signal processing through allowing the actual, natural EM fields to naturally interact in the manner they do in the brain, thereby replicating the same kind of signaling and signal processing (computation). This kind of *in silico* empirical approach is simply missing from the science. No instances of *in silico*-equivalent EM field replication can be found. Artificial neurons created this way could help in understanding EM field expression by excitable cell tissue. It would also facilitate a novel way to test hypotheses *in silico*. Neuroscience and physics, together, could embark on such a development. It would help us reveal the neural dynamics and signal processing that are unknowingly not captured by the familiar models that abstract-away EM fields and that currently dominate computational neuroscience. Note that the computational exploration of the EM fields (*via* Maxwell’s equations) impressed on space by the novel chip would constitute the design phase of the chip. The design would be sent to a foundry to be built. What comes back from the foundry would express the EM fields themselves. The empirical method would be, to neuroscience, what the Wright Brothers’ construction of flying craft did for artificial flight. Thirty years ago, we did not have chip foundries capable of brain-scale (∼5 nm feature size) EM field expression. Now it is routine. With a convergence on EM fields in the science of consciousness, it seems reasonable and apt to begin to explore the potential use of this technique.

### C1 and Some ABC-Correlate Classes in the Modern Science of Consciousness

Loosely guided by the categories of theories found in the Stanford Encyclopedia of Philosophy entry “Consciousness” (Van Gulick, 2018, Section 9) and the Scholarpedia entry “Models of Consciousness” (Seth, 2007), the following six sections examine, under the C1 spotlight, six classes of ABC-correlate theories of consciousness roughly representing how they tend to be found grouped in the science literature.

As far as can be ascertained, and while the classes can be argued to overlap, the list is exhaustive. All ABC not explicitly mentioned seem to fit within one of them. “Active Inference” (Friston et al., 2017), for example, fits into the cognitive/computational class. The “Temporally Integrated Causality Landscape” (Winters, 2020) (TICL), for example, fits into the neurobiological class. And so forth. As an exercise for the reader, a recent major review included eight ABC (Northoff and Lamme, 2020) that might be used as an example. The “correct” ABC-correlate is assumed to be somewhere in the six nominated classes. Exactly which ABC is “right” is moot to the analysis.

Finally, for completeness, we examine, again under the C1 spotlight, ABC = philosophical categorizations as recognized in the introduction. In practice these indirectly and variously map into the six nominated classes and are amenable to our treatment as ABC-correlates under C1.

#### ABC = Neural/Neurobiological Theories of Consciousness

In the first ABC of the modern era, Crick and Koch suggested that “*coherent semi-synchronous oscillations, probably in the 40–70 Hz range*” (gamma synchrony) in primary visual cortex were possibly responsible for aspects of visual experience (Crick and Koch, 1990). Primary visual cortex was later accepted as disproved as a contributor to visual experience generation (Weiskrantz, 1996; Lamme and Roelfsema, 2000; He and MacLeod, 2001; Jiang et al., 2007), but as stated above, this fact is not germane to this analysis. Also included in this ABC category would be the influential “Darwinian neuronal group selection” work of Gerald Edelman and colleagues (Edelman, 1987, 1989, 1992; Edelman and Tononi, 2000).

There are many other interesting contributions in this neurobiological class, too many to list here. Fundamentally, they all distill down to the same approach. Each is an attempt to hold specific neural organization, and its activity, as the originating correlate of some aspect of P-Consciousness. In the process of describing the neurobiological basis, the physics substrate – the EM basis of the tissue – is abstracted away. The C1 perspective tells us, however, that no matter how elaborate the description, or which aspects of the brain are described, or at what descriptive level (cell, cell ensemble, brain region), all are actually implemented as EM phenomena of the Supplementary Material A kind. Posed in this way, their contribution is an implicit enrollment in a form of strong emergence. They lack any principled reason why a 1st-person perspective necessarily inheres in the described tissue behavior and not somewhere else. Such neurobiological accounts of P-Consciousness will, however, get their ultimate connection to P-Consciousness through the EM field system’s delivery of P-Consciousness. This is what C1 tells us about this class of ABC: that the origin of an explanation of a 1PP for the entire class entails the single task of explaining how EM fields deliver the 1PP.

#### ABC = Cognitive/Computational Theories of Consciousness

Cognitive/computational accounts of consciousness involve abstractions (again, the abstracting-away of the EM basis) of brain function that are neuroscience-inspired to an extent determined by the researchers. Cognitive accounts tend to be associated with empirical investigation of function with a focus on a wide range of domains including memory, attention, sensory modalities, motor/actuation systems, language, and so forth. These are applied to a descriptive account of development, learning, intelligence, planning, mood, prioritizing, goal setting, habit establishment, novelty handling, amongst many others. These processes tend to be expressed in information processing terms (McGovern and Baars, 2007).

In approaching P-Consciousness, influential ABC in this class are the “Global Workspace Theory” (GWT) by Baars (1988, 1997) and “Global Neuronal Workspace Theory” (GNW) primarily developed by Dehaene and Changeux (2011), Dehaene (2014), and Mashour et al. (2020). In GWT and GNW, integrated and unified activity of brain regions (such as multiple sensory modalities) is said to “be conscious.” Dehaene and colleagues’ “signatures of consciousness” include high-frequency neural firing synchronization across distant brain regions. Under C1 we can now see that however a “global workspace” might be imagined, the brain implements it as a single unified (global) dynamic EM field system impressed on space. The term “global workspace” is a human abstraction of something comprised of EM fields.

The cognitive approach’s contact with P-Consciousness can be understood in a more general sense in appreciation of the ABC “computation” (generally thought of as “information processing”). When the signal processing or information processing of the brain (such as a “global workspace”) is regarded as computation, it reveals an unusual relationship between nature and models of nature that only exists in brains. Once a particular aspect of the brain’s signal processing is recognized as significant and mentally excised from the tissue for scientific description, the information transformations going on in the abstract model are identical to the information transformations apparently going on in the brain. This relationship between a model and nature is unique to neuroscience. Contrast this with, for example, the “information processing” that is going on in a kidney that results in purified blood. In fire (combustion), it results in heat. These phenomena are not abstract models of something. In the brain, this identity between a model of nature and the modeled nature would indicate that everything the brain does is done by the model. This uniqueness has been pivotal in the impact that computing has had in understanding the brain.

In practice, researchers implement these abstract models on general-purpose (stored-program) computers [digital/von Neumann (Aspray, 1990) or analog/neuromorphic (Schuman et al., 2017)] where there is no fixed relationship between the EM physics of the brain and the EM physics of the general-purpose (GP–) computer. This fact must be remembered when trying to construe any contact between the 1PP resulting from “being” a GP-computer and the 1PP resulting from “being” a brain. If it is held that the GP-computer has a 1PP, then the practitioners have disposed of the specific EM organization of the brain, replaced it with the EM organization of a GP-computer, and enrolled themselves in the same kind of strong emergence discussed above. The implicit claim is that “computation” causes the emergence of the 1PP associated with the original tissue being modeled, but in a way that is not evident in the model. If this approach is not acceptable, then one could abstract out the associated functional role of consciousness into the model. Then the new model might have the 1PP of the modeled nature. Again, the relationship with the origins of the 1PP is strong emergence. No necessary relationship between a 1PP and the EM physics of the GP-computer/model combination is provided by this approach.

The key to understanding this approach’s critical weakness is in the above step “*mentally excised from the tissue.*” At that excision moment, the particular EM field organization of the brain is lost, and that specific loss involves everything that the excised model failed to capture. The way to see this loss more clearly is to ask: “*What is the thing analogous to blood filtration and heat in the above examples that may be lost in the ‘mental excising’?”* What goes missing? How would we know it was missing and justify it? If the original EM included delivery of all the information processing content associated with delivery of a component of a 1PP, then that information is gone and its functional role in the natural process goes with it. That is the loss associated with the novel explanandum that is the 1PP. It is lost in an apparently benign act of mental excision that until now was all there is in neuroscience practice. This is what the ABC = cognitive/computational correlates of P-Consciousness look like under the C1 spotlight: the very thing abstracted away (EM fields organized in the manner of a brain), is the thing delivering (however mysteriously) the 1PP. The practitioners involved cannot claim that nothing is lost in the “abstracting-away” of the EM basis of the tissue. To scientifically examine what is lost is to experimentally retain the natural EM physics for comparison/contrast with the “abstracted-away” version. Successful measurement of the properties predicted by a model does not prove that there are no other important tissue properties at work, where tissue and model may part company in important, interesting ways.

#### ABC = Higher-Order Theories of Consciousness

These ABC are often referred to as metacognition and could possibly be included above in the cognitive/computational class (Rosenthal and Weisberg, 2008; Carruthers, 2018; Van Gulick, 2018; Brown et al., 2019). Where P-Consciousness is an explanandum, these ABC theories focus on an account of the origins of P-Consciousness being exhausted (in terms of necessity and sufficiency) by considerations such as:

(i) The existence of a “self.”
(ii) Agency that “knows that it knows.”
(iii) Representation.
(iv) “Narrative assemblies” of (i)…(iii).

This class of ABC simply holds that these attributes of cognition are the specific properties necessary and sufficient for P-Consciousness to arise. Just as in Information Integration Theory (IIT) (see below), there is substrate-independence in the sense that P-Consciousness emerges in anything (say X) that can be scientifically decomposed into classes (i)-(iv). Substrate independence is challenged by C1. This is because these high-level characteristics, in the end, are also physically delivered by the brain’s EM field-based signaling system that physically implements any/all of (i)-(iv). As already stated here, the human brain, clearly an instance of (i)-(iv) based on EM fields, is the only known originator of P-Consciousness. Once again, in these higher-order approaches, the EM field basis of the actual implementation of (i)…(iv) is abstracted away. This is the sense in which C1 involves itself in this class of theory. They, too, become EM field theories and again engage, in connection to a 1st-person perspective, the explanatory failure that is strong emergence.

#### ABC = “Fundamental” Theories of Consciousness

Some ABC claim to be fundamental in some way, but not in the sense of the standard model of particle physics. This class of ABC-theory of consciousness variously involves new posited characteristics of the underlying structure of the fabric of reality, the usual province of physics, not neuroscience. An early pioneer is Benjamin Libet’s putative “conscious mental field” or “cerebral mental field” (CMF) that “*would not be in any category of known physical fields, such as electromagnetic, gravitational, etc.*” (Libet, 1994, 2006). In pursuit of an explanation of P-Consciousness, Libet, in effect, is implicitly calling for a revision to the standard model of particle physics.

As the science has unfolded, a single, dominant and promising theory of this kind has emerged. It is the “Information Integration Theory (IIT) of Consciousness” by Tononi (2004, 2008), Balduzzi and Tononi (2008), Oizumi et al. (2014), and Tononi et al. (2016). IIT claims that it is the integration of information measured statistically, in terms of mutual information content, that form the necessary and sufficient conditions originating P-Consciousness. IIT proposes that the information content of the system as a whole — over and above the information content of its parts — originates P-Consciousness. In IIT, an undefined microscopic proto-conscious information “mote” is assumed. When this unspecified proto-element is aggregated in the IIT manner, a subject made of the aggregate is claimed to have P-Consciousness of a kind and degree prescribed by the details of the IIT formalisms.

IIT also holds that the physical substrate is irrelevant. From a C1 perspective, this position is rather hard to understand, because C1 tells us there is only one substrate that we know delivers P-Consciousness: EM fields organized in the form of a brain made of atoms. In the formulation of IIT the fundamental EM basis of the brain, the only place known to originate P-Consciousness, is apparently abstracted away. If IIT is claiming it is independent of Supplementary Material A brain EM (atoms), then exactly what other substrate is IIT referring to, and how does it relate to the “information mote” described above? Additionally, nowhere in IIT is there any justified/proved connection, except axiomatically by premise, to why “being” integrated information delivers P-Consciousness and what the fundamental proto-information element might be. A recent variant of IIT suggests that “causal power” is identical to P-Consciousness (Koch, 2019). The primary origin of causation in the brain is that which inheres in its fundamental EM field basis: the Lorentz force (Supplementary Material A). The Lorentz force (EM in general) lacks all specification of “what it is like to be the Lorentz force.” There has, more recently, been some success using EM field measurements to quantify and explore the integrated information content (measured level of P-Consciousness) of the vast and real fundamental EM field system of the brain impressed on space as per Supplementary Material A (Koch, 2019; Seth and Bayne, 2022). How can IIT use EM as empirical evidence (thereby proving EM delivers the 1PP) while, in effect, denying that it is EM that is actually delivering the 1PP? Exactly how does the IIT “information mote” fit into the SMPP in a way that makes sense of this?

For the purposes of this analysis, this kind of “fundamental” ABC also fails as an explanation. Once again, we are left with strong-emergence. IIT, however, fails in a revealing way. IIT implicitly denies C1 (the fact that EM fields deliver the 1PP), replacing it with organizations of an “information mote” that is axiomatically (by fiat) charged with the responsibility for the 1PP. It is in reconciling IIT’s relationship with the (also fundamental) EM field class, that reveals unity in the structure of the science that is the subject of detailed discussion in Part II. Note that presaging this unification are the first explicit encounters between IIT and fundamental physics (specifically EM) (Barrett, 2014; McFadden, 2020).

#### ABC = Quantum-Mechanical Theories of Consciousness

The following two broad categories of QM phenomena form the basis of a potential account of P-Consciousness (Atmanspacher, 2020). First, the “atom” level in the Figure 2B structural hierarchy is stabilized by the interacting (coherent) EM fields expressed by nuclei and electrons being quantized according to QM constraints. Quantized EM field systems produced by the charge content in atoms and molecules are still just EM fields. Down deep in the Figure 2B hierarchy, EM fields themselves are a quantum phenomenon [virtual photon exchange (Jackson, 1999)]. Quantum phenomena are built into the processes of forming molecules from atoms and vice versa. Chemical reactions of all kinds (including enzyme, second-messenger, ligand docking, and ion channel conformation dynamics) are non-equilibrium quantum EM events. “Chemical potentials” are simply electrical potentials within EM field phenomena expressed by atoms and molecules. Heat (thermal radiation) and ultraweak biophotons are also EM field phenomena, again products of QM processes intrinsic to the atomic basis of brains.

Therefore, QM is already built into the substrate (at the Figure 2B [M–3] atomic level) of any EM field treatment of the common matter of our biosphere, prior to any considerations of brain material. It is, therefore, logically entailed that whatever EM fields contribute to an account of P-Consciousness in brains automatically incorporates any QM-constrained affinities operating horizontally, and inherited structural constraints/properties operating vertically within the deep hierarchical structure of Figure 2B.

Second, there is a significant history of attempts at a quantum account of P-Consciousness through attribution to exotic quantum effects within brain structure and activity. “Fröhlich Condensates” and quantum coherences in neuron microtubules are prominent examples of this kind of approach (Fröhlich, 1968, 1970, 1975, 1986; Marcer and Hameroff, 1998; Hameroff and Penrose, 2014). The historical critique leveled at exotic QM accounts of consciousness is that the brain’s high temperature thermodynamics prevents the persistent spatiotemporal coherence (spatial size, intensity, and duration) needed to enable functional relevance (Tegmark, 2000). This critique has not survived. Recent work by various scholars posits strong examples of “warm and wet” quantum coherence in biology, and its involvement in brain tissue can now be taken seriously (Lambert et al., 2013; McFadden and Al-Khalili, 2018). If specialized quantum coherence does happen in the brain (such as the subcellular-level/microtubule exotic QM proposed by Hameroff and Penrose, 2014), it would insert a localized horizontal organizational layer in the EM hierarchy of Figure 2B at the tissue (M+1) level.

Broadly speaking, in either of these two categories of QM, wave functions constrain EM fields. QM-constrained EM fields are, however, still EM fields. Note that a proven absence of QM-constrained coherence in EM fields at the functional level in excitable cell tissue does not exclude the possibility that classically constrained coherence in EM fields operates at the same functional level. Macroscopic coherence through intermittent EM field resonances could therefore originate P-Consciousness merely through the quantum mechanics that already pervades the Figure 2B hierarchy. Either way, it is again supra-atomic EM fields that proximately deliver P-Consciousness and its dynamics.

Under the C1 spotlight, we can now see that *quantum mechanics is actually part of an EM field theory of P-Consciousness*, but the atomic-level EM basis of QM propositions tends to be lost in the process of explication of the QM details.

Note that an ABC theory of P-Consciousness that extends its attribution of P-Consciousness origins to properties of the subatomic layers (including the other three quadrants of the standard model) does not invalidate the EM basis of P-Consciousness. It is the EM fields that carry forward the subatomic level activity/properties to the higher levels in the Figure 2B nested containment hierarchy. This is a consequence of the natural containment hierarchy’s reframing of P-Consciousness as a product of the (EM) unity of the entire hierarchy.

#### ABC = Electromagnetic Field Theories of Consciousness

Electromagnetic field theories of P-Consciousness have their own long history but tend to present sparsely and rest in relative obscurity. For example, a recent major review focused on eight ABC while not mentioning EM fields as a basis for consciousness, even though it is represented within the eight (Northoff and Lamme, 2020). Another recent major review covered four classes and thirteen individual ABC also completely lacked attention to EM fields as a basis for consciousness (Doerig et al., 2020). As this article goes to press a new review article has been published listing 22 theories of consciousness, including the EM field theory of consciousness. This evidences a small improvement in the visibility of the EM account of consciousness (Seth and Bayne, 2022). The abstracting-away of the EM basis of the brain (physics-shyness within neuroscience) is a common factor that is the most likely explanation of the observed relative obscurity. Modern-era pioneers of EM field theories start with Sue Pockett in the 1990s (Pockett, 2000). This was followed early in the century by McFadden (2002a,b, 2006, 2007, 2013, 2020). Later, we have a contribution by (Fingelkurts et al., 2013). For reviews, including the early history and its pioneers, see Jones (2013, 2017) and Pockett (2013).

A recent example is the General Resonance Theory (GRT) of consciousness (Hunt and Schooler, 2019), which offers a general theory that encompasses mammalian/vertebrate consciousness and any other species of consciousness, whether that consciousness is based on EM fields or any other kind of field. GRT focuses on the Oscillatory Correlates of Consciousness (OCC), where the particular “oscillations” most relevant to P-Consciousness are those arising from the brain’s endogenous EM field system as described in Supplementary Material A.

The abovementioned EM account offered by JohnJoe McFadden is the wave-mechanical approach in his “Conscious Electromagnetic Information” (CEMI) field theory (McFadden, 2002a,b, 2006, 2007, 2013, 2020). “*I therefore examine the proposition that the brain’s EM field is consciousness and that information held in distributed neurons is integrated into a single conscious EM field: the CEMI field*” (McFadden, 2002a). In essence, it is the information content of the wave-mechanical behavior within the spatial structure of the brain’s endogenous EM field that is claimed to deliver P-Consciousness.

The abovementioned (Fingelkurts et al., 2013) is a result of earlier developments that ultimately became “Operational Architectonics” (OA). It specifically describes P-Consciousness as arising in the complexity of a system of nested EM fields of the kind described in the section on containment hierarchy and in Supplementary Material A.

Paradoxically, C1 tells us that the EM class of theory also fails to explain the 1PP and leaves us with an explanatory gap. EM fields do not come pre-packaged (within the existing standard model) with an explicit, principled scientific account of “what it is like to be EM fields”. Because of this, EM fields formally fail to explain P-Consciousness. Therefore, at first blush, the various EM accounts also relate to P-Consciousness in the strongly emergent manner of any other ABC. However, EM fields are fundamental, and for this reason, they inherit a way forward in fundamental physics tackled later in Part II.

#### ABC and Philosophy in the Explanation of P-Consciousness

At this moment in its relatively nascent development as a physical science, philosophical analysis can still sometimes form a part of a scientific approach to explaining P-Consciousness. There is one significant form of this in play at the moment: panpsychism (Skrbina, 2007; Chalmers, 2016; Goff et al., 2018). It offers an interim way to deal with the refractory lack of ultimate explanation of P-Consciousness in any ABC-correlate. Panpsychism operates as an approach of the “fundamental” class, where a novel field or particle or similar elemental component/property of a 1PP (akin to charge or spin) is considered built into the underlying fabric of the universe in some way. In effect, the unspecified property panpsychism invokes is something extra, invisibly inherited and accreting along the Figure 2B vertical EM field hierarchy.

Like fundamental ABC, this approach implicitly invokes a connection to a missing or incomplete part of the standard model of particle physics. It can be used in combination with an ABC to notionally complete its contact with a full explanation of P-Consciousness, thereby avoiding strong emergence. Used like this, panpsychism acts as a placeholder that does the job of recognizing that explanation is missing without requiring immediate attention to the lack of a scientific law of nature (*within* the standard model) that defines what the superadded fundamental element is and how it functions. This is how GRT and IIT connect to their conception of P-Consciousness, thereby explaining it *via* the normal weak emergence within a *future* standard model (although neither speak of it in standard model terms). In this way, panpsychism operates as an explanation-in-waiting for a later upgrade to the standard model that scientifically solves the hard problem. Such a potential future upgrade is outlined below in Part II.

The above analysis of panpsychism is likely to be typical of the many philosophical treatments of subjectivity. That is, under C1, the philosophical ABC also fail/succeed as a form of correlate, although in nuanced ways (as exemplified for panpsychism) that are best left to philosophers to properly calibrate. Note that EM field theories of consciousness have no obvious philosophical category umbrella that we can cite with any authority, and if we were able to do so, it would not alter any of the outcomes of this article. Like all ABC, the philosophical ABC will also get their ultimate contact with explanation through the physics/neuroscience collaboration focused on EM fields.

#### ABC Classes and C1: Conclusion

A few salient features of the analysis are:

• Both the ABC = QM class and the ABC = EM class are fundamental in the sense of the existing standard model of particle physics.
• The fundamental class IIT is fundamental in the sense that it addresses the fabric of reality, but outside the existing standard model.
• The ABC = QM class is revealed as an EM field theory and should be considered inside the EM class.

Overall, the analysis depicts how each class connects to the explanatory failure mode of “strong emergence” in a slightly different way that the C1/EM field approach has the potential to redress. *However, the EM field class of ABC fails in the same way*. That is, P-Consciousness arises for reasons that are not delivered by merely nominating “EM fields behaving ABC-ly.” The EM field basis of the 1PP, proved under C1, does not transform any ABC into explanation of the 1PP. C1 merely locates where the solution is to be found. In effect, we are left with two fundamental classes, IIT and EM, both failing to deliver real explanation in the manner described. However, because they are “fundamental,” they have a potential route to explanation afforded by their fundamental physics status. That potential is to be explored in Part II.

### Concluding the Case for C1

The hierarchical view tells us that the familiar complex endogenous EM field system of the brain is not a side effect produced by excitable cells made of something else. The entire thing *is* electromagnetic fields, from the atomic level up (impressed on space with atomic level resolution). What we normally encounter in excitable cell tissue is merely the final, net observable expression (in an EM field signal-to-noise sense) of a natural system entirely comprised of nested (Figure 2B) EM phenomena organized in Supplementary Material A form.

A neuron is a collection of EM fields “behaving neuron-ly” to an observer made of EM fields. Terms like “chemical,” “chemical reaction,” “chemical pathway,” “electricity,” “electro-chemical,” “chemical potential,” “action potential,” “electrical/chemical synapse,” “Nernst potential” and many other recognizable terms used to distinguish cellular processes and properties, are not pointing to anything other than an EM field system behaving in a certain way. “Electrical current” is a transit of an EM field system through space. That transiting EM field system (magnetic and electric) is impressed on space by the transiting charge source. The EM basis of the tissue applies deep down into the substructure of atoms, where quantum mechanics is merely a set of (wave-equation-based) quantizing constraints on EM field expression. This is the kind of readjustment that is necessitated when drawing a connection between EM fields and P-Consciousness. Which of the many candidates is the “ABC” activity that originates the 1PP? Whatever it is, the ABC’s ultimate contact with explanation of a 1PP inheres in the EM basis of all ABC because an ABC is actually a descriptor delineating particular aspects of an EM field system. It does not matter if the ABC involves descriptions of information content, information processing, signal processing, energy transformations, networking, anatomical details, causality, entropy, function, or anything else. The descriptive scale (subcellular/atomic, cell, cell ensemble or cell population) of an ABC does not matter. No matter how elaborate or technically abstract the ABC, it is physically implemented as an EM field system of the kind exemplified in Supplementary Material A.

As a result, and however mysterious it is to us, a hierarchy of the fundamental physics of electromagnetism based on atoms somehow defines the context of the human brain’s origination of both its outward (3rd-person-perspective or 3PP) observable behavior and its 1st-person perspective (1PP). Under C1 we can now see that to explain P-Consciousness involves more than merely specifying an ABC. It also involves an additional account of how “being” electromagnetism delivers a 1PP. This is because EM field is literally what we are made of. With our current understanding of weak/strong emergence, it is our ultimate task here to curate the circumstances under which the 1PP may ultimately become a weakly emergent (predictable) property of a future, deeper understanding of EM field activity.

Even without an explanation of how EM fields originate the 1PP, an EM field account of P-Consciousness is intrinsically advantaged and has much to commend it. This occurs merely because of the well-understood properties of EM fields and their fundamental physics status. Under the C1 dialogue we have seen that it is (for reasons not provided yet) EM fields, configured in ABC form, that actually deliver the computation/signal processing/information flow behind cognition while simultaneously delivering P-Consciousness, but only when the EM fields are configured in the special ABC way (whatever that turns out to be). This easily explains how unconscious brain signaling processes can arise that are also entirely made of EM. In unconscious brain process, the normal signaling (also made of EM) continues to act in the familiar adaptive manner but lacks a contribution to the 1PP because it does not incorporate the extra specific EM structure/dynamics of the necessary ABC kind. It has also been shown how EM has intrinsic natural solutions to the unity, binding, grounding and combination problems while providing for P-Consciousness to involve itself in the causality inherent in the fundamental physics of the brain (see Supplementary Material A and the Lorentz force as well as Kitchener and Hales, 2022). An EM perspective also naturally handles time. Contents of consciousness can enter and exit P-Consciousness (addition and removal of a particular vectorial contribution to the EM field system) with a variable spatial/temporal granularity and at the rate of the field system dynamics, with the observed levels of continuity/discontinuity, and with the subtle experiential “flavor” of the passage of time (Kent and Wittmann, 2021). Under C1 we have also seen that from a measurement and control perspective, neuroscience has tacitly been enrolled in an EM account of P-Consciousness all along. All the evidence collected in the science of any ABC is also acting in direct support of an EM field theory. These are the advantages all ABC inherit through C1 and the relocation of the science of P-Consciousness into the fundamental physics of the existing standard model.

### Part I Final Result: Summary

This section compiles the first of two overall results from this article that form the basis of our “electromagnetic turn” in the science of consciousness:

1. Through C1, we now understand how 30 years of ABC-correlates science has delivered an enormous body of evidence that the standard model’s EM quadrant delivers the 1PP (by means not specified). All correlates of consciousness are actually electromagnetic correlates.
2. The science undergoes a shift in emphasis involving a convergence (for everyone involved) on EM fields as the ultimate origin of the 1PP. EM fields are moved to center-stage in the science of consciousness.
3. The science is formally connected with fundamental physics. This is because (i) EM fields are a quadrant in the standard model and (ii) EM fields, through the nested hierarchy shown in Figure 2B, literally connect fundamental physics directly to a neuroscience context, spanning the entire interdisciplinary gap. The future therefore necessarily involves a close collaboration between physics and neuroscience. This connection is highlighted by the Figure 2A line C. It is a transdisciplinary connection consistent with the unification of the brain in the nested EM field hierarchy shown in Figure 2B. The responsibility for science’s account of P-Consciousness is to be shared.
4. One of, or a combination of, the many existing ABC will be right (the “right correlate”). Nothing in what has been delivered here denies that. What is denied by C1 in the above analysis (in the unique, unprecedented context of the 1PP as an explanandum), is that delivery of the “right” ABC-correlate also delivers explanation. This is the explanatory (strong emergence) cul-de-sac identified in the introduction: in the absence of prior knowledge of the underlying mechanism of a 1PP, the low likelihood of empirically proving that the “right” ABC has been found.
5. The use of the empirical results arising in the use of the above, *in silico* generation of the brain’s EM signaling physics, is also flagged as a potential activity for the new neuroscience/physics collaboration.

These five changes provide a solid basis for the science to progress into its fourth decade and beyond. However, we must also recognize that the changes to the conduct of the science are posed while being fully cognizant that none of them deliver the reasons why/how EM fields have, within them, the potential for a 1PP and what specialized form EM fields necessarily take when delivering it. Having used the standard model to arrive at this point, we must also encounter the paradoxical fact that there is nothing in the standard model’s EM quadrant that specifies “what it is like to be EM fields.” We can take some solace in the knowledge that we have focused the ultimate source of the problem (lack of explanation) to one location in fundamental physics. Dealing with the lack of explanation is the subject of the next section.

## Part II: A Speculated Route to Explanation for the Science of P-Consciousness

The Part I result stands on its own as a way forward. We could have stopped there. It involved a reframing of perspective that shifted the explanation of the origins of the 1PP to a single place in fundamental physics: Electromagnetism. That is, the “where to look” part of explaining P-Consciousness is solved. What is not solved is how EM can be re-examined/reframed in a way that somehow reveals “what it is like to be” EM fields. This is the moment when the real challenge is laid bare: the uniqueness of the explanandum. How do we introduce, into science, a way of dealing with the 1PP? We can proceed with one key new bit of knowledge: that a way of introducing a novel explanandum exists in the 1PP of electromagnetic fields. Neuroscience has proved EM fields can create a 1PP. It is now up to us to explore how an explanation of the 1PP of EM fields can be approached. In what follows, the most important factor is *that there appears to be at least one way ahead*. It is not fully articulated and is posed as a tentative exploration. It is in this possibility that we hope that we can escape the “strong emergence cul-de-sac.” The challenge is in the realization that the shift in thinking is a shift in how we organize *ourselves* as scientists. It is a “discovery” about the operation of science itself. It should not be surprising that a new kind of scientific explanandum necessitates some kind of reframing or expansion of our options for scientific behavior. The 30 years of the modern form of the neuroscience of consciousness give us the latitude to explore this possibility so that a discussion can be taken up, forming a nucleus of activity for the neuroscience/physics collaboration to come.

We start by reaffirming what was found in Part I: the proved EM field basis of the 1PP does not transform any ABC into explanation of the 1PP. C1 merely locates where the solution is to be found. Here in Part II, we move forward by recognizing that explanation involves a separate fundamental physics account, of an as yet unknown kind, of how EM fields deliver a 1PP, thereby adding explanation (underlying or bottom–up mechanism) to the science of P-Consciousness, potentially transforming the strong emergence to weak emergence, normalizing the science of subjectivity in the sense of the section on weak/strong emergence. The following discussion does not “solve the hard problem.” It merely locates a suggested departure point of a trajectory that offers the best hope of it. It delivers, in fundamental physics terms, the origin of a potential account of the 1PP that clearly somehow inheres in EM fields. In doing so, and because of C1, all ABC-correlates theories benefit equally, and the “right” ABC correlate can, in the end, be empirically confirmed conclusively. The remarkable aspect of what follows is that it naturally merges IIT (Integrated Information Theory) into the EM class, locating them both in fundamental physics, *but in a revised standard model* that procedurally offers a route to finding the missing explanation of *why/how* EM field, configured in the form of the “right” ABC, delivers the 1PP.

The starting point is the consequence of C1 found in Part I summary result 1: that the 30 years of work on ABC-correlates has, in effect, delivered a vast body of evidence that the Figure 1 SMPP’s EM quadrant can and does deliver a 1PP to human brains. This creates a direct encounter with a deep anomaly: the current form of the SMPP lacks any account of the 1PP (subjectivity) of any member of its four quadrants in any context. “*What is it like to be an EM field?*” has no answer. Likewise, the possibly irrelevant but nevertheless possible question “*What is it like to be a neutrino?*” has no answer. Yet neuroscience tells us that the SMPP’s EM quadrant delivers a 1PP. This anomaly is demonstrating the incompleteness of the SMPP as an explanatory instrument. This is the doorway to a way ahead, just as anomalous scientific evidence has been so many times in science (Kuhn and Hacking, 2012). Clearly the SMPP is missing whatever kind of scientific account of nature is needed to explain the 1PP proven to be delivered by one of its quadrants.

Remember that our natural nested containment hierarchy approach has already revealed the generalized “1PP-voicelessness” that currently pervades the whole of science. The 1PP-voicelessness results from the presupposition of the 1PP in the form of the scientific observer that accessed and provided all the evidence that validates laws of nature of the kind currently produced by science. Scientific behavior’s generalized critical dependence on the 1PP for its evidence source, in effect, means that scientific behavior, as it is currently configured, can scientifically describe, and in some sense explain, everything in the universe except the 1PP (the scientific observer) it presupposes. If scientific behavior is regarded as a completed or somehow fixed behavior (no justification for this has been found in the literature, it is simply presupposed), then this situation could be regarded as “game over” for a scientific account of the 1PP. But now we have new evidence – the SMPP anomaly – that we can examine with a view to potentially overcoming this limitation. The approach explored here is that scientific behavior itself is incomplete and is in need of revision in some sense.

Before we tackle the SMPP anomaly, we need to better understand the “critical dependence” of scientific behavior on the 1PP. It is something that tends to be invisible in science. Understanding it properly is part of the key to understanding how, for example, the SMPP can be expanded to accommodate the explanation of the 1PP currently lacking in it. As already noted in Part I, in seeking the goal of the science of consciousness, the science of the 1PP is implicitly and ultimately being used to explain the nature of the acquisition of any/all “objective” scientific evidence, by *any* scientific observer. In the current 3rd-person-perspective (3PP or “objectively evidenced”) mode of the operation of science, at the end of the evidence trail in every finished act of scientific measurement, we scientists *demand* that the contents of the consciousness of a scientific observer (say, S), during an encounter with the measurements themselves or their representational proxy, becomes a formal part of the evidence inference trail that empirically proves every 3PP law of nature (such as F = mA or the existing SMPP, for example). This final step of passing measurements through the 1PP of scientific observer S is or completes an act of “scientific observation” by S. Without the involvement of that final stage of evidence acquisition, involving the natural causality that somehow originates the 1PP within the brain of the observing scientist S, applied to the chain of evidence, scientist S cannot claim to have “objective evidence” in support of any 3PP hypothesis. Counterevidence, also delivered *via* the 1PP of the scientific observer S, is similarly demanded to refute or modify 3PP laws of nature. We scientists insist that such “contents of consciousness” be experienced, documented and *repeatable* by other scientists (the contents of the 1PP of different and various S) on pain of having our scientific claims rejected in critical argument through lack of scientific evidence. Put another way, if it weren’t for the 1PP (subjectivity) of the scientific observer S, there would be no “objectivity.” Put yet another way, the apparently objectively evidenced 3PP laws of nature are actually predicting how nature shall appear in the 1PP of a presupposed scientific observer. Moreover, without the 1PP of scientist S, creating 3PP laws of nature would be a meaningless concept because there would be no (scientific) observer to experience, as contents of the 1PP, the predicted observable consequences of a studied/hypothesized natural regularity.

This critical dependency of 3PP laws of nature on the observer’s 1PP is, at a surface level, at odds with our sense of the “observer independence” that objectivity is supposed to bring to the process of creating 3PP laws of nature. What we call disciplined “objectivity” clearly and successfully works to render 3PP laws of nature independent of the 1PP of any *specific* human scientific observer S. However, the achieved “specific-human-scientific-observer-independence” cannot be used to claim the 3PP laws are independent of (invariant to) the specific physics of the generation of the human 1PP itself. This is not the first time this has been noted (Rosen, 1993)^1^. In being required to coerce nature’s regularities into a form suited to engagement with the 1PP of a scientist, the 1PP itself, however benignly, is imprinted on the observed nature. The 1PP is, in this way, implicitly built into all 3PP laws of nature.

Now consider what happens when the familiar “objective” 3PP evidence process, with its demonstrated critical dependency on the 1PP, is applied to construct a science of the 1PP itself: the science of P-Consciousness. In that context the science of P-Consciousness, with its unprecedented and unique explanandum, the 1PP, operates at a scientific evidence “boundary condition” – the explanation of the scientific observer that no other science inhabits and in which it is not the contents of the 1PP that are being explained, but the very existence and nature of the 1PP itself.

This is the constellation of unique circumstances that surround the critical dependency that our system of establishing 3PP laws of nature has on the 1PP of its presupposed and consequently unexplainable scientific observer. With this understanding of the critical dependency in hand, we now return to the anomaly identified earlier when the 1PP-voicelessness of the SMPP confronts powerful evidence from neuroscience that the SMPP’s EM quadrant does indeed deliver the 1PP.

The strong anomaly, visible only across the Figure 2A extent of line C in the context of C1 within the science of P-Consciousness, spanning the physical sciences into the fundamental physics of the EM quadrant of the SMPP, gives physicists, under the guidance/empirical support of neuroscience, license to explore whatever kind of novel abstract description of nature has the potential to reveal the origins of the subjectivity that is empirically proved (by neuroscience) to inhere in EM fields. Let us first designate as (i) the familiar 3PP class of scientific “laws of the appearance of nature” (such as those that form the existing SMPP). Our physics/neuroscience collaboration, inspired by the anomaly, can now set about creating a novel kind of abstract description of nature, say type (ii), that somehow *does* explain the 1PP. Clearly these new (ii) laws of nature *cannot presuppose the scientific observer* in the manner of the existing (i) 3PP laws. Instead, the (ii) descriptions must somehow assist in *predicting* the scientific observer and in the process explain the origins of the 1PP that creates the possibility of (i) 3PP laws of nature. This basic idea is the main contribution of Part II. Notice that this change presents as a change in ourselves as scientists. The options for scientific behavior have been expanded to allow a new, categorically distinct, kind of abstract scientific description of nature. Laws of nature of an as yet unspecified kind (ii).

What is it that the new set (ii) of abstractions of nature are describing? The manner of the failure of the existing SMPP to predict the 1PP gives us the main clue. The failure presents as proof of a stark difference between (i) what the universe *appears* to be made of, and what the universe is *actually* made of. The SMPP anomaly tells us these two things cannot be the same. This is highly suggestive that the new (ii) descriptions must somehow depict laws of the underlying structural fabric of the universe in a way that is very different to (i) but yet is consistent with and ultimately somehow predictive of how it appears in (i). The difference between (i) and (ii) descriptions, and their simultaneous mutual consistency in describing the same natural world in two different ways [(i) appearance and (ii) underlying structure], offers a route to understanding the mechanism that creates the 1PP of the scientific observer presupposed by (i).

We can use the SMPP as a vehicle to bring this bifurcation of the abstract products of scientific behavior into a more practical light. The current SMPP is a product of the current confinement to (i) 3PP laws of nature. Our proposition is that the standard model’s scope of scientific deliverables, and the scientific behavior that produces them, is to be expanded to include (ii). We now know that EM field, as depicted by the particular (i) 3PP “laws of appearances” in the SMPP’s EM quadrant, is merely a (i) appearance of something behaving EM-field-ly to a scientific observer apparently, but not actually, made of EM fields. Instead, the scientific observer is made of something else. What is this building block of the underlying fabric of reality described by (ii)? At this point we must be mindful that the full elucidation of the (ii) new kind of descriptions of nature is well outside the scope of this article. This is a job for a physics-neuroscience collaboration. What we can do here is make a few general observations about descriptions (i) and (ii) before we sign off.

To help tease out the difference between (i) “appearance” descriptions (what the universe, say, U, appears to be made of, such as “space,” “atoms” or “EM fields”) and (ii) “underlying structure” descriptions (what the universe U is actually made of), let us assume that (ii) involves abstractions describing a universe made of a large collection of a single kind of primitive structural element, say X. This “X” could be perhaps regarded as an “event” or “information mote” or “energy quantum” or all these simultaneously. Its true identity is not our job to specify here. We do this to emphasize the point that under the proposed upgraded standard model’s science framework, the scientific observer inhabiting the Figure 2B hierarchy at layer [M+3], is *actually made of X*. Indeed, the entire Figure 2B hierarchy is actually made of X. What the hierarchy *appears* to be made of, to a scientific observer made of X and located within the hierarchy, is EM fields emerging from the depths of the nested containment hierarchy depicted in Figure 2B. That is, the Figure 2B hierarchy is merely how the hierarchy appears to an observer embedded in, and part of, a hierarchy actually made of X. It is the web of causal relations between instances of X that literally creates the hierarchy. It is in the underlying structure of a web of causal relations between X in the context of their literal creation of what we see as EM fields operating in a brain context, that we can find the origins of the 1PP. The upgrade to the operational structure for science, driven by a need to explain the origins of the 1PP, is instead leading us to the origins of causality in nature that have been mysterious for centuries (Hume and Steinberg, 1993). The two problems can now be seen as correlated in a manner to be explored in the new (ii) “laws of underlying structure” necessitated by the science of P-Consciousness.

Next we can summarize the final state of the upgraded framework for science, perhaps best understood as a description of the “natural world of human scientific behavior” in the following three contexts:

(i) Abstract “Laws of Appearances” constructed by a scientific observer S inside our universe U. The descriptions are based on scientific evidence that arrives in S as the contents of the consciousness (1PP) of S. This produces the familiar 3PP (“objectively evidenced”) models of nature that predict how U appears (regularities evident in what U appears to be made of) to a presupposed scientific observer S. For example, S is the presupposed scientific observer that acquired all the evidence that proved the existing SMPP. The existing SMPP is a system of analytic (mathematical) laws of kind (i).

(ii) Abstract “Laws of the underlying structure of a U made of X.” These abstractions are also constructed and explored by scientist S. The process results in a categorically distinct class of analytically or computationally/algorithmically explored abstract formalisms that depict the underlying fabric of reality as a collection of networked structural primitives X. The structural primitives have nothing directly to do with space or atoms or EM fields or any other (i) 3PP “laws” and are of a kind exemplified below. Hidden within the explored systems of networked abstractions of X we will find (with the correct abstract X and appropriate computational or analytic metrics and their probes) emergent properties of U we recognize as space, atoms, EM fields and (ultimately) the scientific observer S inside U, also actually made of X, that is mandated to “see” U operating in the manner of the (i) laws of nature. Within (ii) we have an account of the origins of the 1PP that explains the scientific observer that simultaneously provides equal empirical proof of both the (i) and (ii) abstract scientific accounts of U by S.

(iii) The actual universe U, made of what we have abstracted as X in (ii), that has a real scientific observer S in it who has a real 1PP in which “contents of the 1PP” originate all the scientific evidence supporting both the (i) “laws of appearances” and the (ii) “laws of underlying structure.” Note that the *in silico* chip discussed in Part I is *empirical* science exploring (iii) to validate (i) and (ii) in the same way that, say, cellular organoids or flying aircraft are exploring (iii). Abstract descriptions (i) and (ii) are the “real” *theoretical* science products (delivered into the journal system within U) of a human scientific observer S. Do not confuse (i) and (ii) abstract descriptions of U, and/or their exploration with general-purpose computers (also made of X), with the (iii) actual U.

The solution to the hard problem, we suggest, has been hard because it must be discovered (not invented) in a completely different realm of descriptions of nature of kind (ii). In effect, the very meaning of what it is that a scientist does to explain nature has itself had to change.

What scientific evidence do we have that it is possible or practical to describe the natural world U in (ii) form? When we look for it, we easily find that we have already been doing it (X descriptions) for decades, *but in physics and outside the science of consciousness*. They are familiar to all of us. Some examples: X = “string theory” e.g. (Sen, 1998), “loops” e.g. (Rovelli, 2006), “branes” e.g. (Ne’eman and Eizenberg, 1995), “dynamic hierarchies of structured noise” e.g. (Cahill and Klinger, 1998, 2000; Cahill, 2003, 2005), “cellular automata” e.g. (Mitchell et al., 1994; Hordijk et al., 1996; Wolfram, 2002), and “quantum froth” e.g. (Swarup, 2006).

Additionally, and relatively recently, *within the science of consciousness*, it is possible to reinterpret the “Information Integration Theory (IIT)”-correlate of consciousness as being a contributor of (ii) descriptions of X (*via* its specified X = “information mote”). The kinds of networked abstract-X descriptions can easily be recognized in IIT, for example see Albantakis and Tononi (2017). That being the case, we already have an example of X and (ii) *within the science of consciousness* – it just hasn’t been formally recognized in “standard-model-upgrade” terms that physics can accommodate. It is a connection to EM that provides the mechanism for accommodating IIT into fundamental physics. Working with physics to reformulate IIT in (i)/(ii) terms is probably the best way to launch a revision to the operational framework of science in the form of an upgraded SMPP. It would powerfully validate and enable IIT, allowing it to migrate to its proper place within a revised standard model on the other side of the disciplinary/explanatory gap. It seems an apt way of forging a path ahead. IIT and its variants have already demonstrated unique progress in “detecting consciousness” *using EM field measurements* (Koch, 2019; Seth and Bayne, 2022). It may be that IIT’s apparent access to deeper insight is actually a result of it being unknowingly involved in the novel (ii) kind of fundamental physics. The science framework upgrade approach explains why IIT has had so much trouble proving its validity under the current science framework e.g. (Merker et al., 2021). It has had to artificially erect an entrance to (ii) in the form of the many postulates (axioms) upon which its proposals are based. This article offers the potential to replace the postulates with empirically proved fundamental physics (*via* the EM field basis of the 1PP) and thereby deliver a route to the empirical support it needs. If neuroscience proposals like IIT require the addressing of matters relating to the fabric of reality (however they do this), the correct place to do so is in its natural home, across the transdisciplinary divide in fundamental physics, not in neuroscience.

Having created this system of epistemologically dual-aspect [paired, appearance/underlying structure, (i)/(ii), 3PP/1PP] abstract, symbolic scientific descriptions of nature, empirically proved in an account of the 1PP (*via* the EM field basis of the scientific observer), the practical form of a trajectory toward a solution to the hard problem exists as follows: Motivated by the essential knowledge that EM somehow delivers the 1PP, computational and/or analytic mathematical investigation of (ii) self-modifying tangled webs of X are conducted. They explore the various X such as strings, loops, branes, structured noise hierarchies, cellular automata, quantum froth, “*IIT information motes”* and so forth. They are explored to see if they can be configured in a manner that naturally expresses emergent processes that can be interpreted to have the properties we recognize as space, atoms, charge/spin systems and so forth, expressing EM fields of the familiar (i) kind. The moment a (ii) collection of abstracted X can be found to express EM fields as an emergent behavior of the collection, the physicists involved, by directly comparing the (i) and (ii) depictions of the same nature, would then be able to see, within (ii), that part of the underlying structure of (i) that may be responsible for the 1PP. That may then suggest a fundamental principle that would apply if a 1PP was to somehow be a result of the difference between (i) and (ii). That principle, it is proposed, is either the ultimate solution to the hard problem or a route to it. Posed as a possibility within a revised framework for science, the principle is something to be discovered, not invented. We authors do not know what this principle is, but we look forward to somebody discovering it.

The revised dual-aspect standard model/framework for scientific behavior is intrinsically self-evidencing because of its capacity to account for the scientific observer. All the evidence that proved all the (i) familiar laws of nature is also brought to bear in evidencing (ii). The content of the existing (i) standard model is unaffected by the additional set of (ii) descriptions (they are a categorically distinct class). Rather, (i) forms a set of well formulated and time-tested constraints that can be used to find and formulate the correct set (ii) descriptions. Once the set (ii) descriptions are established and can naturally express EM as an emergent property, and EM’s role in creating the brain’s 1PP is understood, all ABC-correlates proposals have the means to validate/invalidate their claim to have captured the “correct” correlate of the 1PP. Neuroscientists will then know what to look for in the brain to find the delivery-sites of the 1PP, in all its kinds and degrees.

### Part II Final Result: Summary

Until the above approach is used, the formal lack of explanation is predicted to continue to thwart the ABC-correlates paradigm indefinitely. Indeed, even if the perfect “smoking gun” ABC-correlate is somehow located and proved, the researchers involved would still be left high and dry wondering why/how the 1PP arises and would end up with a need to seek the kind of ultimate explanation process depicted above. That process will lead to the “discovery” of the full extent of scientific behavior, the lack of which possibly underlies the confounds that have prevented progress for so long. It is hoped that our Part II speculations start a dialogue directed at developing these ideas into solid proposals. IIT would be a recommended place to commence that dialogue because it is already involved in the shift, albeit informally, inadvertently and incompletely. To assist, a final reminder of the two existing consolidations of IIT and EM: (Barrett, 2014; McFadden, 2020). It is hoped that the above analysis has helped to extend these propositions in the service of all theories of consciousness (all ABC). EM is ultimately at the heart of the matter for everyone. Researchers familiar with EM, and that see EM’s role as obvious, may find this article helpful in bringing EM into the territory of the bulk of researchers that traditionally have little or no awareness of EM (Kitchener and Hales, 2022), but that are now critically dependent on it as the ultimate source of explanation for their own theories of consciousness.

## Conclusion

In pursuit of a solution to the decades-long struggle we all inhabit in turning abstract observational correlates into cogent explanation supported by a fundamental principle, this article reframes the science of P-Consciousness through its relocation into the relatively foreign land (to mainstream neuroscientists) of EM fields. It is based on the empirical fact that it is EM fields that ultimately deliver P-Consciousness. This is something that is as empirically certain as it is uncertain exactly how they do it. The correlates of P-Consciousness paradigm must ultimately face the fundamental physics of EM fields if a fully explanatory account of P-Consciousness is to be constructed. The necessary physics-neuroscience collaboration involved in this “electromagnetic turn” pushes EM fields to explanatory center-stage in the science of consciousness, a location that has also been demonstrated to have at least some potential to take us a little closer to a solution to the “hard problem”.

## Author Contributions

CH contributed the first draft of the manuscript. CH and ME contributed to manuscript revision, read, and approved the submitted version. Both authors contributed to the article and approved the submitted version.

## Conflict of Interest

The authors declare that the research was conducted in the absence of any commercial or financial relationships that could be construed as a potential conflict of interest.

## Publisher’s Note

All claims expressed in this article are solely those of the authors and do not necessarily represent those of their affiliated organizations, or those of the publisher, the editors and the reviewers. Any product that may be evaluated in this article, or claim that may be made by its manufacturer, is not guaranteed or endorsed by the publisher.

## Footnotes

1 Observer independence is not always achieved. Quantum mechanics has shown us this is not always possible, as the Rosen article details.
