# Supplementary material for: Electromagnetism’s Bridge Across the Explanatory Gap: How a Neuroscience/Physics Collaboration Delivers Explanation Into All Theories of Consciousness
Source: Front Hum Neurosci. 2022 Jun 16;16:836046. doi: 10.3389/fnhum.2022.836046 (PMC9245352; doi:10.3389/fnhum.2022.836046)
Supplement: Supplementary file 1 [file Data_Sheet_1.pdf]

# Electromagnetism’s Bridge Across the Explanatory Gap

## Supplementary A

### The ultra-scale origins of excitable cell tissue-level EM

by
Colin G. Hales and Marissa Ericson

What is the form of the endogenous EM field system expressed by excitable cells? It is well-known that it is cranial central nervous system networked excitable cell activity that delivers P-Consciousness in humans (Crick, 1994; Koch, Massimini et al., 2016; Tononi, Boly et al., 2016). But exactly how does it do this? Here we demonstrate how C1 and EM field knowledge impact the way we describe brain tissue EM field origins in a manner that reflects its potential role in the origins of P-Consciousness. Note that in the EM field’s delivery of P-Consciousness, it delivers an additional advantage in that the causality inherent to the fundamental physics of EM fields (Vector superposition and the Lorentz force) provides the potential for an intrinsic, although indirect, connection between the EM field and a role for P-Consciousness in influencing behavior.

Historically, the endogenous EM field system of the brain has been regarded as epiphenomenal — a causally inert incidental byproduct such as the sound produced by a heart. However, outside of considerations targeting P-Consciousness origins, a causal (modulatory/adaptative) role for the brain’s endogenous EM field system is recently and increasingly finding empirical support. This self-modulating phenomenon in excitable cell tissue is a form of EM field coupling typically referred to as ‘ephaptic coupling’ or ‘ephaptic transmission’ (Anastassiou and Koch, 2015; Anastassiou, Perin et al., 2011; Chiang, Shivacharan et al., 2019; Frohlich and McCormick, 2010; McFadden, 2020; Qiu, Shivacharan et al., 2015).

In assessing theories of consciousness from a C1 perspective, care must be taken to explicitly distinguish between how EM fields may modulate neural signaling dynamics in general, their role in delivery of P-Consciousness and how these may interrelate (how consciousness may acquire a causal role in brain tissue). Proponents of any given ABC may or may not pay attention to causality. Causality tends to be sidelined above the main article Figure 2A line B boundary where the ABC descriptions progressively lose contact with the cellular physics basis of the tissue.

We now examine how C1 and the Figure 2B ‘vertical view’ reveals the EM field systems at the layer [M+1] tissue level. At the Figure 2B layer (M) cell/neuronal level, the established dynamic signaling systems of the brain are action potentials (somatic and dendritic, membrane-longitudinal) and the EM-field coupling described above (membrane-transverse). Both these EM field signaling types are simultaneously caused by the same system of membrane-embedded sources. A third signaling type, again from membrane-embedded sources, is the chemical synapse that also generates a dynamic EM field in the process of contributing to the potential triggering of the first two signaling types.

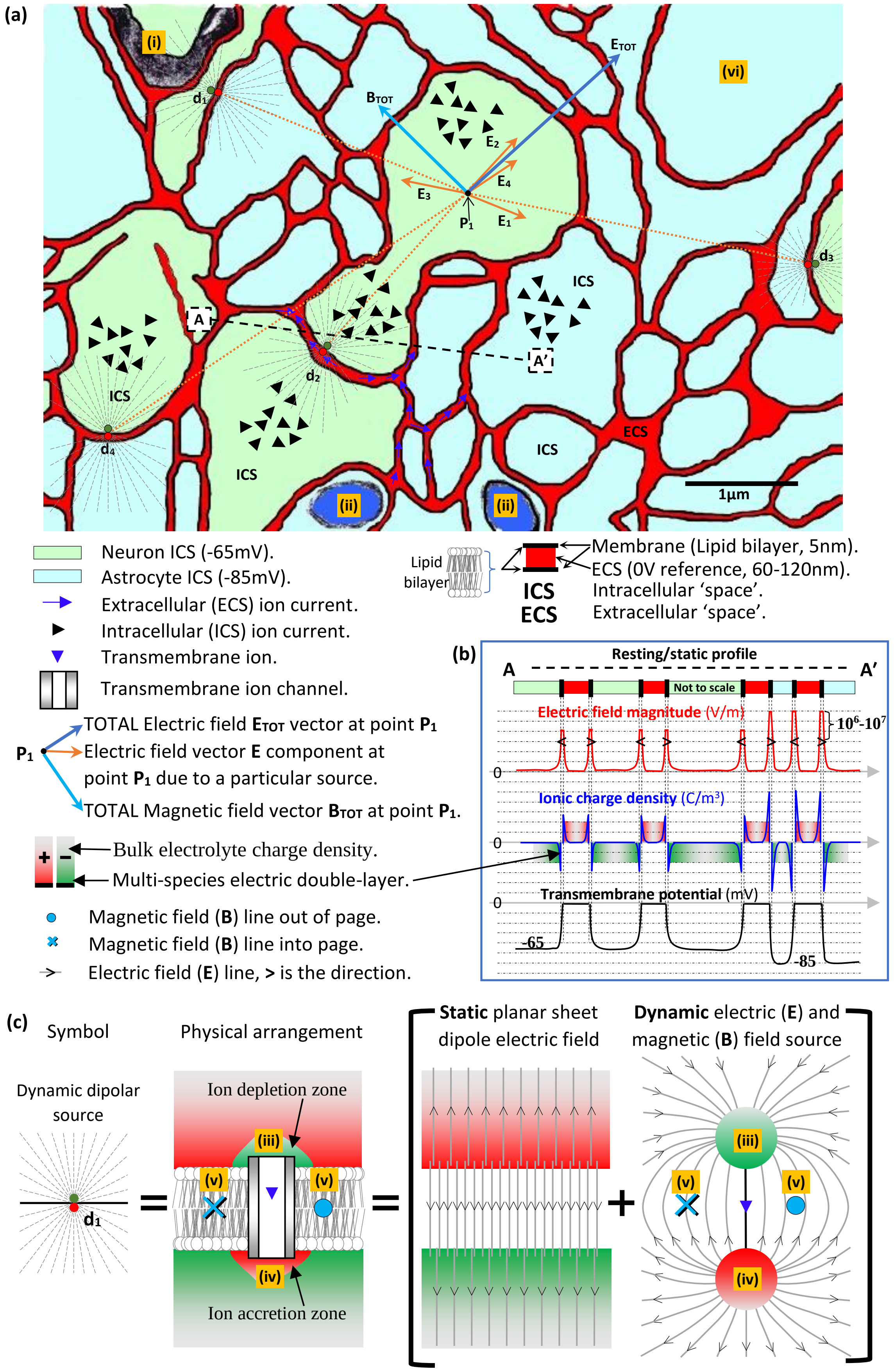

(a)
(i)
(vi)
(ii)
d1
d2
d3
d4
BTOT
ETOT
E1
E2
E3
E4
P1
ICS
ECS
A
A'
1μm
Neuron ICS (-65mV).
Astrocyte ICS (-85mV).
Extracellular (ECS) ion current.
Intracellular (ICS) ion current.
Transmembrane ion.
Transmembrane ion channel.
Lipid bilayer
Membrane (Lipid bilayer, 5nm).
ECS (0V reference, 60-120nm).
Intracellular 'space'.
Extracellular 'space'.
TOTAL Electric field ETOT vector at point P1
Electric field vector E component at point P1 due to a particular source.
TOTAL Magnetic field vector BTOT at point P1.
Bulk electrolyte charge density.
Multi-species electric double-layer.
Magnetic field (B) line out of page.
Magnetic field (B) line into page.
Electric field (E) line, > is the direction.
(b)
Resting/static profile
Not to scale
Electric field magnitude (V/m)
10^6-10^7
Ionic charge density (C/m^3)
Transmembrane potential (mV)
0
-65
-85
(c)
Symbol
Physical arrangement
Dynamic dipolar source
Ion depletion zone
Ion accretion zone
(iii)
(iv)
(v)
Static planar sheet dipole electric field
Dynamic electric (E) and magnetic (B) field source

**Figure A.1** The ultra-scale grey-matter origins of the endogenous electric field **E** and magnetic field **B** systems. Based on (Nicholson and Sykova, 1998), (a) depicts the tissue context of the EM field origins. (i) is an instance of myelinated axon from the original micrograph. Similarly, (ii) are mitochondrial cisterns. These are pointed out for completeness. (b) depicts the membrane source origins of the static **E** field. (c) depicts the membrane source origins of dynamic **E** and **B** fields. The coloring of (iv) red and (iii) green is the electrical dipole equivalent of the identification of the north (outward-flowing) and south (inward-flowing) magnetic poles of, say, a bar magnet dipole. (v) depicts the field magnetic field lines that circulate the transmembrane current. For further explanation see the main text.

It is the EM fields of these three different signaling contexts that superpose to become the observed dominant endogenous EM field system of the tissue. A biologically realistic depiction of the nanometer-scale origins of the EM field system is shown in Figure A.1(a). Figure A.1(a) is based on a manually stylized electron micrograph of a slice of cat cortex neuropil (Nicholson and Sykova, 1998). Figure A.1 is a summary of the systems of well understood brain signaling electromagnetism sources as reported, from an EM field perspective (Hales, 2011; Hales, 2014; Hales, Grayden et al., 2011; Hales and Pockett, 2014) and the many articles and standard texts referenced therein, in particular those related to Maxwell's equations. Everything commonly described as 'electrochemical' in dynamic signaling processes in somatic, axonal, dendritic and synaptic contexts, are included in the Figure A.1 depiction of EM field phenomena.

Figure A.1(a) is entirely a result of electromagnetism impressed on space by layer (M-2) atoms. At the [M+1] tissue level depicted in Figure A.1(a), a pair of fundamental fields is produced: a single vector electric field (**E**) and a single vector magnetic field (**B**), each of which pervade the entire tissue and emerge into the space outside the tissue. There is no empty space in the tissue. Dominating these fields is the contribution, by vector superposition, of the collective action of millions of the same kind of Figure A.1(c) field sources that exist at the nanometer scale of ion channels embedded in cell membrane. These contributions dominate the tissue spatially (size/extension), temporally (duration) and in intensity. This EM field signal activity drowns out all the remaining EM field noise produced by the atoms of the rest of the cellular chemistry.

Figure A.1(c) is an abstraction of an ion-channel-based EM field source system capable of originating the three above kinds of signaling. It is shown decomposed into two field system components. On the left is a source of static **E** field system. On the right is a dynamic component that self-modulates the static **E** field, thereby producing a dynamic **E** field system and a dynamic **B** field system.

## 1 Static Sources (A) and (B)

This is a source of electric **E** field only. It exists in two places in the brain in large quantity: inside all (A) neuron cell membrane and all (B) astrocyte cell membrane. This is a huge, tortuous area of highly charged membrane. The transmembrane charge partitioning system produces a large, inwardly directed (ECS=>ICS) static electric field (**E**) across the cell membrane's lipid bilayer. In Figure A.1(a)/(b) this static **E** field exists within the black lines that are the external cell membrane of all cells. These lines extend into and out of the page. Any individual cell has of the order $10^5 \mu m^2$ of membrane. Any individual cell shown in the image may extend this static **E** into and out of the page up to a potential distance of tens, hundreds or even thousands of times the size of this micrograph in all directions. This is the convoluted surface along which action potential signaling travels 'membrane-longitudinally'. This membrane-centric static sheet **E** field is described at the micron-scale in Figure A.1(b) as a profile along the Figure A.1(a) line A-A', with the static **E** field established in and around the (black) cell membrane by ionic charge sources. Its actual form is shown at the ultra-scale level of detail in Figure A.1(c), as the left (static) component of a (static/dynamic) decomposition of the fundamental field source created by a single ion channel embedded in the membrane.

These huge static **E** fields (A) and (B) operate in the origination of the dynamic component of the **E** and **B** phenomena, acting as a kind of EM field 'canvas' upon which all EM field dynamics are 'painted' by ion channels. It is the static transmembrane quasi-planar electric field 'sheet' dipole across the entire outer cell membrane of all neurons and astrocytes when charged up to their resting potential (≈-65mV for neurons and ≈-85mV for astrocytes). At around $10^7$ V/m (Freeman, 1975; Lee, Klingler et al., 1994; Maggio, Borioli et al., 2008; Peterka, Takahashi et al., 2011; Pethig, 1986; Romijn, 2002), the static transmembrane **E** field is the most intense field expressed at a spatial scale larger than any of the atomic/molecular components that work to erect and maintain it (including ion transporters, translocators and pumps not shown in Figure A.1). Exposed to atmosphere, electric fields of this intensity would be a sparking hazard in typical macroscopic electrical hardware. This electric field is also spatially huge in area. Based on a rough knowledge of the total number of cells and the cell membrane surface area of astrocytes and neurons in normal, healthy adult human cranial brain tissue, it adds up to thousands of square meters. This field exists as an exquisitely thin (5nm) sheet of powerful electric field tortuously impressed on this vast area of space by the black membrane shown in Figure A.1(a).

# 2 Dynamic Sources (C), (D) and (E)

These are the fundamental unit source of the dynamic component of the total EM field responsible for the above three familiar brain signaling processes. Dynamic **E** and **B** both arise in the same signaling-related collapse and restoration events within (A) neuron membrane. They do not arise in astrocyte (B) membrane. This punctate source dipole mechanism is depicted in Figure A.1(c) as a combination of the static (A) electric sheet dipole and a single ion channel's dynamic activity that simultaneously creates both a membrane-transverse dynamic **E** electric dipole (coordinated in colocalized plaques of different ion channel types, these sources can actually cause a localized reversal in transmembrane polarity during an action potential) and a membrane-longitudinal dynamic **B** field originating (circulating) inside the plane of the membrane. Both **E** and **B** arise as a result of transmembrane (ion-channel) current flow. Collections of collocated adjacent ion channels act in both spatial and temporal unison (coherence) and when their electric and magnetic fields vectorially superpose, they simply create a larger, more intense dynamic dipole. As previously discussed, this Figure A.1(c) activity happens in three source contexts labeled (C), (D) and (E) as follows.

## 2.1 Source (C): Synapses.

This is the neuron membrane-bound post-synaptic ion channel plaque that includes chemical/neurotransmitter-gated ion channels. These plaques can occur anywhere on the cell membrane of neurons, but mostly occur in the dendrite processes on dendritic spines customized to purpose. This situation exists in Figure A.1(a) in the source dipole symbol $\mathbf{d}_2$ on the post-synaptic density of a real dendritic spine. Note that the transmembrane current behavior in this context can create a local Figure A.1(c) dipole that does not reverse direction. This is the mechanism by which a single ion channel deposits a persistent quantum of excitatory or inhibitory charge into the post synaptic cell, thereby contributing to graduated cell potential activity that may or may not result in an action potential triggered by the (D) and (E) source systems.

## 2.2 Source (D): Dendritic action potential (DAP) propagation sites.

These are a relatively recently described 'firing' triggered by (C) that, if a dendrite's local membrane ion channel plaques are distributed appropriately, may cause a saltatory dendritic action potential to propagate (see (E) for more detail). It can then travel into and spread through the dendrite structure to the extent permitted by the distribution of other dynamic ion-channel plaque sources in the dendrite structure (Gidon, Zolnik et al., 2020; Jia, Siegle et al., 2019). The (D) propagation front travels at roughly one m/s, tortuously along the membrane.

### 2.3 Source (E): Somatic axon potential (SAP) propagation sites.

This is the action potential activity triggered by an ion channel plaque in the neuron soma. The plaque is usually located at the junction of a soma and its axon process and is called the 'axon initial segment' or 'axon hillock'. The action potential propagation is again supported by appropriately placed ion channel plaques (dynamic EM field source dipole(s)) acting as 'repeaters'. It travels in this 'saltatory' manner both dromically down the axon and anti-dromically across the soma and out into any dendrite processes supported by the necessary ion channel 'repeater' placement. In axons these repeater stations are the 'nodes of Ranvier', a cylindrical zone of collected dynamic sources in between sections of myelination. Dromic (E) axonal saltatory signaling, at roughly one hundred m/s, is the fastest membrane-longitudinal signaling wave speed in the tissue. The anti-dromic propagation of the same (E) action potential travels at roughly one m/s, tortuously along the membrane, and its propagation front can collide with dendritic (D) source activity.

### 2.4 (C), (D) and (E) sources: overview

EM field sources (C), (D), and (E) can be thought of as originating in localized clusters of cooperating Figure A.1(c) transmembrane ion channels (called plaques above). When they operate, they create a dynamic **E** and **B**. First, an ion channel's transmembrane current projects a brief, weak **E** field influence out into the tissue, extending membrane-transversely at distances greater than the 5nm thick intense static field that is being disrupted. Second, it projects a brief, weak, circular **B** field influence membrane-longitudinally, circulating around the ion channel transmembrane current, at distances greater than the size of the plaque that generated it. Both **B** and **E** field decrease in intensity at a rate proportional to the cube of the distance from the source system. The best way to imagine the Figure A.1(c) dynamic source component is to imagine that when an ion channel pore is shut it looks like a piece of non-conducting plastic. When an ion channel pore opens, no matter how stochastically, its unitary, brief conduction of a few picoamperes conducts like it was traveling through an extremely thin, straight copper wire. This copper analogy is a strong one. The transport speed of the charge carriers (ions) is actually comparable in magnitude to that found in copper, but only during ion channel transit. This process has been compared to the operation of a field-effect transistor (Bezanilla, 2005).

## 3 How sources create the endogenous EM field

The total endogenous **E** and **B** field is a vector summation of the contributions of every single Figure A.1(c) membrane-embedded ion-channel source. In Figure A.1(a), the field contributions from the ICS current (black arrowheads) and ECS current (blue arrows) rapidly de-correlate and diminish into EM noise and contribute nothing to the endogenous **E** and **B**. ICS and ECS ions travel at a drift rate orders of magnitude slower, and randomized in direction, compared to when they were in the source transmembrane channel. Maxwell's equations tell us that it is current *density* that creates an EM field contribution, not current. While the ECS and ICS total current reflects its ion channel origins, in the ECS and ICS their contribution to a current *density* vanishes in comparison with their current density while in transit through the membrane. Their contribution to the overall field system goes with it. Therefore, ECS and ICS currents can be regarded as irrelevant as an overall **E** and **B** source, while participating in the equilibration of the localized ICS and ECS charge density depletion and accretion disruptions caused by ion channel traffic. These disruptions are shown as Figure A.1(c)(iii) and (iv), respectively.

Vector superposition of each and every ion channel's **E** and **B** field source contribution sum to create a single EM field whole with collective dynamics. For illustrative purposes, four single-channel source systems of the Figure A.1(c) kind have been randomly placed in the (notional) neuron cell membrane in Figure A.1(a), shown as $\mathbf{d}_1$, $\mathbf{d}_2$, $\mathbf{d}_3$ and $\mathbf{d}_4$. There may be hundreds of these sources collocated at each of the four locations, cooperating temporally, in which case

the intensity of their 'signal', as a single EM voice, is increased through spatial and temporal coherence.

Next, consider a single field observation point in space: $\mathbf{P_1}$. Both the **E** field and the **B** field from every source pervades all the space around it. Each source impresses its own influence on $\mathbf{P_1}$ directly, at the speed of light. Even if the locale containing $\mathbf{P_1}$ was empty space (vacuum), the EM field would be impressed on the point $\mathbf{P_1}$. At point $\mathbf{P_1}$, sources $\mathbf{d_1}$...$\mathbf{d_4}$ deliver **E** and **B** field vector contributions $\mathbf{E_1}$...$\mathbf{E_4}$ (shown) and $\mathbf{B_1}$...$\mathbf{B_4}$ (not shown). These independently add vectorially in space to produce a single **E** and **B** field depicted as $\mathbf{E_{TOT}}$ and $\mathbf{B_{TOT}}$. Combining these sources with all the other myriad Figure A.1(c) sources within line of sight, creates the unified, complex and dominant EM field that we call 'the endogenous EM field' of excitable cell tissue. The way $\mathbf{E_{TOT}}$ and $\mathbf{B_{TOT}}$ arrow/vectors behave is to move between periods of low intensity noise (a short vector, rapidly reorienting to point randomly) to periods of structure where the intensity increases, and the vector grows and orients itself in a more systematic trajectory as the various contributing components wax and wane in direction and strength.

We are not able to measure this nano-scale field with current technology. The smallest sensor ever placed in tissue is bigger than the ICS region (vi) in the top right-hand corner of Figure A.1(a). A large penetrating electrode, damaging and displacing sources as it is pushed through the tissue, creates a microenvironment of its own. It would destroy the synapse containing source $\mathbf{d_2}$. Measurements of **E** and **B** in ECoG, EEG and MEG are done by sensors typically 10 to 100 times the size of the entire Figure A.1(a) image. The EM field is impressed on space with ion-channel-plaque-level spatial and temporal regularity that we cannot measure, creating intricate patterns in space over and above, in 3D, the EM field noise that 'is' the underlying chemistry.

In an EM field origin story of P-Consciousness, this is the single unified EM field we, as human beings with brains comprised of neurons and glia, are 'being'. It includes all sources (A), (B), (C), (D) and (E) simultaneously, as an expression of fundamental physics.

Let us now move our observation point $\mathbf{P_1}$ to the location of $\mathbf{d_3}$ and consider all the field contributions, $\mathbf{E_{TOT}}$ and $\mathbf{B_{TOT}}$, from every source except those of $\mathbf{d_3}$ and its local plaque neighbors. $\mathbf{E_{TOT}}$ and $\mathbf{B_{TOT}}$ at point $\mathbf{d_3}$, 'exogenous' to $\mathbf{d_3}$, may influence its behavior as a source. It may alter the local firing threshold, thereby advancing or retarding the moment of 'firing' of an action potential triggered at the location of $\mathbf{d_3}$. It may also do something to any contribution $\mathbf{d_3}$ makes to P-Consciousness.

Note that the 'depletion' and 'accretion' phenomena depicted in Figure A.1(c) originate the **E** field membrane-transverse influence. In the normal operation of tissue, and additionally, because of asymmetry in the ECS and ICS bulk electrolyte volumes and with continuous cell firing activity, the 'depletion' and 'accretion' zones can become large and persistent. This accounts for the large-scale (layer-to-layer) slow electric dipoles observed in cortex (e.g. the up/down cycles in (Frohlich and McCormick, 2010)). Large-scale average **B** field (measured by MEG) activity can also be predicted to emerge, for example, from cortical tissue in the collective action of millions of **B** field sources vectorially superposing in space as action potentials travel in coarsely spatially and temporally aligned membrane processes (e.g., parallel dendritic trunks).

Note that the apparent weakness in strength of **E** and **B** ion channel sources is not a reason to discount their contribution to P-Consciousness. For an **E** and **B** source to be enrolled in P-Consciousness, it must merely be sufficiently large (in intensity, and extension and duration)

to allow it to integrate itself, by vector superposition, into the local unity of the full structure of the endogenous EM field.

## 4 References

Anastassiou, C.A., and Koch, C. (2015). Ephaptic coupling to endogenous electric field activity: why bother? Current opinion in neurobiology 31, 95-103.

Anastassiou, C.A., Perin, R., Markram, H., and Koch, C. (2011). Ephaptic coupling of cortical neurons. Nature Neuroscience 14, 217-223.

Bezanilla, F. (2005). Voltage-gated ion channels. IEEE Transactions on NanoBioscience 4, 34-48.

Chiang, C.-C., Shivacharan, R.S., Wei, X., Gonzalez-Reyes, L.E., and Durand, D.M. (2019). Slow periodic activity in the longitudinal hippocampal slice can self-propagate non-synaptically by a mechanism consistent with ephaptic coupling. The Journal of Physiology 597, 249-269.

Crick, F. (1994). The Astonishing hypothesis : the scientific search for the soul (London: Simon & Schuster).

Freeman, A.J. (1975). Mass action in the nervous system : examination of the neurophysiological basis of adaptive behavior through the EEG (New York: Academic Press).

Frohlich, F., and McCormick, D.A. (2010). Endogenous Electric Fields May Guide Neocortical Network Activity. NEURON 67, 129-143.

Gidon, A., Zolnik, T.A., Fidzinski, P., Bolduan, F., Papoutsi, A., Poirazi, P., Holtkamp, M., Vida, I., and Larkum, M.E. (2020). Dendritic action potentials and computation in human layer 2/3 cortical neurons. Science 367, 83-87.

Hales, C.G. (2011). Doctoral Thesis: The Electric Field System of an Excitable Cell. Dept of Electrical and Electronic Engineering, University of Melbourne https://minerva-access.unimelb.edu.au/handle/11343/36741.

Hales, C.G. (2014). The origins of the brain's endogenous electromagnetic field and its relationship to provision of consciousness. Journal of Integrative Neuroscience 13, 313-361.

Hales, C.G., Grayden, D.B., and Quiney, H.M. (2011). The Electric Field System of a Macular Ion Channel Plaque. In 33rd Annual International Conference of the IEEE Engineering in Medicine and Biology Society (EMBC 2011) (Boston, Mass. USA).

Hales, C.G., and Pockett, S. (2014). The relationship between local field potentials (LFPs) and the electromagnetic fields that give rise to them. Frontiers in Systems Neuroscience 8, 233.

Jia, X., Siegle, J.H., Bennett, C., Gale, S.D., Denman, D.J., Koch, C., and Olsen, S.R. (2019). High-density extracellular probes reveal dendritic backpropagation and facilitate neuron classification. Journal of neurophysiology 121, 1831-1847.

Koch, C., Massimini, M., Boly, M., and Tononi, G. (2016). Neural correlates of consciousness: progress and problems. Nature Reviews Neuroscience 17, 307-321.

Lee, K.Y.C., Klingler, J.F., and McConnell, H.M. (1994). Electric-Field-Induced Concentration Gradients in Lipid Monolayers. Science 263, 655-658.

Maggio, B., Borioli, G.A., Del Boca, M., De Tullio, L., Fanani, M.L., Oliveira, R.G., Rosetti, C.M., and Wilke, N. (2008). Composition-driven surface domain structuring mediated by sphingolipids and membrane-active proteins. Cell Biochemistry and Biophysics 50, 79-109.

McFadden, J. (2020). Integrating information in the brain’s EM field: the cemi field theory of consciousness. Neuroscience of consciousness 2020, 13.

Nicholson, C., and Sykova, E. (1998). Extracellular space structure revealed by diffusion analysis. Trends in Neurosciences 21, 207-215.

Peterka, D.S., Takahashi, H., and Yuste, R. (2011). Imaging Voltage in Neurons. NEURON 69, 9-21.

Pethig, R. (1986). Ion, Electron, and Proton Transport in Membranes: A Review of the Physical Processes Involved. In Modern Bioelectrochemistry, F. Gutmann, and H. Keyzer, eds. (New York: Plenum Press), pp. 199-239.

Qiu, C., Shivacharan, R.S., Zhang, M., and Durand, D.M. (2015). Can Neural Activity Propagate by Endogenous Electrical Field? The Journal of Neuroscience 35, 15800-15811.

Romijn, H. (2002). Are virtual photons the elementary carriers of consciousness? Journal of Consciousness Studies 9, 61-81.

Tononi, G., Boly, M., Massimini, M., and Koch, C. (2016). Integrated information theory: from consciousness to its physical substrate. Nature Reviews Neuroscience 17, 450.
